# Image scanning microscopy based on multifocal metalens for sub-diffraction-limited imaging of brain organoids

**DOI:** 10.1038/s41377-025-01900-3

**Published:** 2025-10-13

**Authors:** Yongjae Jo, Hyemi Park, Seho Lee, Hyeyoung Yoon, Taehoon Lee, Gyusoo Bak, Hanjun Cho, Jong-Chan Park, Inki Kim

**Affiliations:** 1 Department of Biophysics, Institute of Quantum Biophysics, Sungkyunkwan University, Suwon, Republic of Korea; 2 Department of Intelligent Precision Healthcare Convergence, Sungkyunkwan University, Suwon, Republic of Korea; 3 Center for Quantum Technology, Korea Institute of Science and Technology (KIST), Seoul, Republic of Korea; 4 Department of Biopharmaceutical Convergence, Sungkyunkwan University, Suwon, Republic of Korea; 5 Department of MetaBioHealth, Sungkyunkwan University, Suwon, Republic of Korea

**Keywords:** Metamaterials, Imaging and sensing

## Abstract

Image scanning microscopy (ISM) is a promising imaging technique that offers sub-diffraction-limited resolution and optical sectioning. Theoretically, ISM can improve the optical resolution by a factor of two through pixel reassignment and deconvolution. Multifocal array illumination and scanning have been widely adopted to implement ISM because of their simplicity. Conventionally, digital micromirror devices (DMDs)^1^ and microlens arrays (MLAs)^2,3^ have been used to generate dense and uniform multifocal arrays for ISM, which are critical for achieving fast imaging and high-quality ISM reconstruction. However, these approaches have limitations in terms of cost, numerical aperture (NA), pitch, and uniformity, making it challenging to create dense and high-quality multifocal arrays at high NA. To overcome these limitations, we introduced a novel multifocal metalens design strategy called the hybrid multiplexing method, which combines two conventional multiplexing approaches: phase addition and random multiplexing. Through numerical simulations, we demonstrate that the proposed method generates more uniform and denser multifocal arrays than conventional methods, even at small pitches. As a proof of concept, we fabricated a multifocal metalens generating 40 × 40 array of foci with a 3 μm pitch and NA of 0.7 operating at a wavelength of 488 nm and then constructed the multifocal metalens-based ISM (MMISM). We demonstrated that MMISM successfully resolved sub-diffraction-limited features in imaging of microbead samples and forebrain organoid sections. The results showed that MMISM imaging achieved twice the diffraction-limited resolution and revealed clearer structural features of neurons compared to wide-field images. We anticipate that our novel design strategy can be widely applied to produce multifunctional optical elements and replace conventional optical elements in specialized applications.

## Introduction

**Fig. 1:**
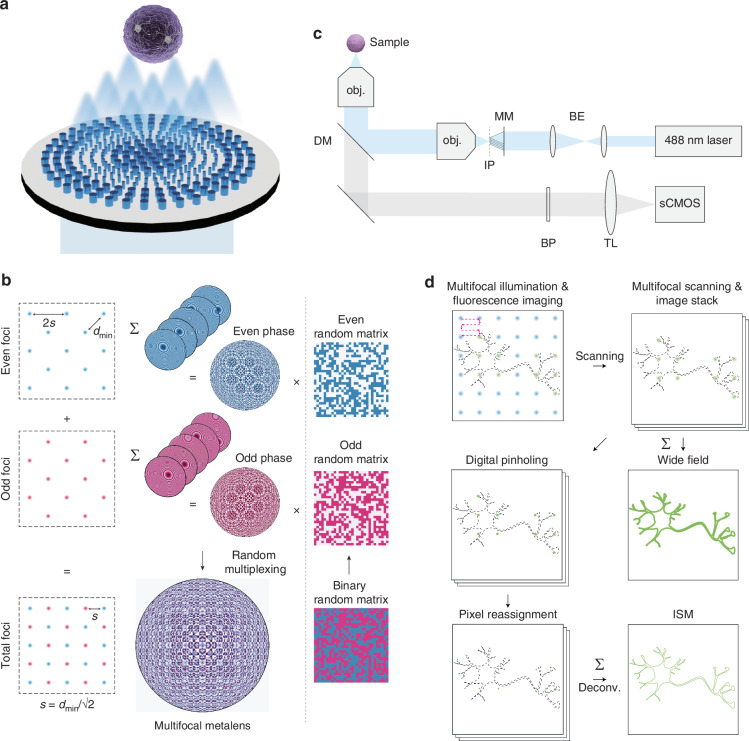
**Overview of the MMISM.** **a** An illustration of the multifocal metalens. **b** Schematic representation of the hybrid multiplexing method for designing the multifocal metalens. Hybrid multiplexing is a novel method that combines conventional phase addition and random multiplexing techniques, resulting in dense and numerous multifocal arrays. The odd and even phase maps are generated using phase addition method, then both phase maps are integrated through random multiplexing to minimize coherent interference between foci. The *d*_min_ represents the minimum pitch between adjacent foci for either the even or odd foci. The pitch (*s*) of the multifocal metalens is smaller than *d*_min_. **c** An illustration of the MMISM setup. MM multifocal metalens, IP intermediate image plane, BE beam expander, DM dichroic mirror, BP bandpass filter, TL tube lens, obj. objective lens. **d** Workflow of data acquisition and image processing for MMISM. Deconv. means a deconvolution

Image scanning microscopy (ISM) is a super-resolution imaging technique that doubles resolution through pixel reassignment and deconvolution^2–8^. Theoretically, confocal detection enhances the optical resolution, which improves with smaller pinhole^9^. However, reducing the pinhole size decreases the light collection, leading to a lower signal-to-noise ratio (SNR)^10^. ISM is an alternative technique that provides enhanced resolution and an optical sectioning effect without significant SNR loss by adopting an array detector where each pixel functions as a tiny confocal pinhole. ISM has evolved into a promising technique offering practical resolution improvements and compatibility with other imaging techniques. Among the various implementations^1,2,4,11,12^, the scanning of multifocal arrays is the most common approach (Fig. 1a). In practice, the creation of a highly dense and uniform multifocal array is crucial for achieving high-speed ISMs with a large field-of-view (FOV). Conventionally, digital micromirror devices (DMDs) and microlens arrays (MLAs) have been used to generate multifocal arrays. However, DMDs are not cost-efficient and require complicated alignments, making it difficult to construct and maintain the setup. Although MLAs are relatively cost-effective and easy to use, they have limitations in enhancing a numerical aperture (NA) greater than 0.6 and reducing the pitch to less than 100 μm due to manufacturing constraints^13–17^. Furthermore, it is challenging to control the pitch and NA of MLAs independently, which requires significant demagnification to achieve a high-resolution and dense multifocal array that could potentially lead to aberrations.

To address these limitations, we introduced a metalens, which is a flat optical element consisting of nanostructures that enables extraordinary optical modulation. Several types of multifocal metasurfaces have been reported, such as Dammann gratings or metalens arrays. However, the Dammann grating suffers from low NA (<0.4), difficulties in pitch adjustment, nonuniform intensity, and a limited number of foci^18–20^. Additionally, metalens arrays have physical limitations in terms of sub-lens size and the number of meta-atoms^21,22^. Since the pitch of the focal spots is directly determined by the sub-lens size, reducing the pitch decreases both the light collection per sub-lens and the number of meta-atoms comprising each sub-lens. The small number of meta-atoms makes the focal quality highly susceptible to fabrication defects. Moreover, the small sub-lens limits the high-NA capability, as the focal length could potentially become smaller than the axial resolution, making it physically unachievable. One effective way to create multifocal arrays is the simple addition of phase profiles. We refer to this as the phase addition method throughout this paper^23^. Multifocal metalens can be realized by integrating the multiple phase profiles of metalenses using this phase addition method. However, in this method, reducing the pitch is restricted by the interference between adjacent foci. Another conventional approach is random multiplexing, which is widely used to combine multiple holograms^24–27^. Multifunctional metalens can be designed using this method by combining phase profiles that are multiplied by a binary random matrix. Although this method is less sensitive to interference between adjacent foci, it degrades the quality of the foci as the number of integrated phase profiles increases, because multiplication by the random matrix impairs each phase profile.

Here, we introduce a novel multiplexing strategy called hybrid multiplexing, which combines two conventional methods to leverage their advantages. First, we separated the target multifocal array into two staggered multifocal arrays, termed even and odd foci, to increase the distance between the adjacent foci, thereby preventing interference (Fig. 1b). The phase profiles creating even and odd multifocal arrays were generated using the phase addition method. The phase profiles were then combined using random multiplexing. This approach integrates the phase profiles with reduced interference while minimizing the degradation in the beam quality because only two random matrices are used (Fig. 1b). We conducted wave propagation simulations with various parameters and concluded that the proposed method enables a more uniform multifocal array with a smaller pitch and higher NA than conventional design methods. To validate the simulation, we fabricated a silicon nitride (SiN) multifocal metalens operating at 488 nm designed to generate 40 × 40 foci with a pitch of 3 μm and NA of 0.7. Furthermore, we demonstrated that the pitch can be further reduced using polarization modulation. Because orthogonally polarized light does not interfere with each other, adopting polarization modulation enables a denser multifocal array, which we refer to as the polarization hybrid multiplexing method.

As a proof of concept, we constructed a multifocal metalens-based ISM (MMISM) using the proposed multifocal metalens (Fig. 1c). We demonstrated that MMISM beat the diffraction limit by imaging microbeads and organoid tissue samples. MMISM imaging achieved a resolution of ~330–370 nm in full width at half maximum (FWHM), nearly twice that of the wide-field (WF) microscope (~590–720 nm) at NA of 0.5, as determined by imaging 0.03 μm fluorescent beads. Furthermore, we obtained the MMISM images of neurons in 40 μm thick forebrain organoids stained with MAP2 and pTau antibodies^28^, which visualize the complex neuronal skeletal structures as they target microtubule-related proteins^29–31^. Notably, the confocal rejection capability of MMISM, achieved through digital pinholing process, enables the scattering removed clear images even in thick tissue samples, which are not attainable with a WF microscope. In the field of neuroscience, observing the fine details of neuronal structures in thick scattering tissue is critical for unveiling the functionality of the nervous system. Compared to WF microscope images, MMISM provides enhanced resolution and optical sectioning effects, displaying clearer features of the neuronal fiber structures. Furthermore, MMISM successfully resolved sub-diffraction-limit fine features separated by ~330–370 nm, whereas the WF microscope could not distinguish these structures. We believe that our hybrid multiplexing method offers a promising strategy for implementing novel optical modulation applications, such as the proposed MMISM.

## Results

### Image processing for multifocal illumination ISM

In fluorescent imaging, structured illumination microscopy (SIM) is widely used for super-resolution imaging, achieving twice the resolution of the diffraction limit using structured light. The maximum resolution of SIM is determined by the sum of the spatial frequencies measurable by the detection system and generated by the illumination pattern. Notably, ISM is a special case of SIM that uses a diffraction-limited focal spot as a structured light, incorporating all spatial frequencies allowed by the illumination NA^4^. Like SIM, ISM has various implementations and reconstruction algorithms. Among them, we will introduce multifocal illumination ISM to demonstrate the feasibility of our novel multifocal metalens.

Multifocal illumination ISM achieves resolution doubling through several processes, including multifocal illumination and seamless scanning, digital pinholing, pixel reassignment, and deconvolution (Fig. 1d). First, multifocal illumination and scanning generate image stacks containing fluorescent focal spots. Digital pinholing computationally applies apertures to the center of fluorescent focal spots to reject out-of-focus signals^8,32^ (Fig. S1). This step provides critical advantages in removing scattering and background noise, which are unavailable in conventional WF microscopy. Pixel reassignment improves resolution by a factor of $\sqrt{2}$ by placing the digitally pinholed focal spot patches onto the higher-resolution image with doubled pixel density while maintaining their original locations. Finally, deconvolution further enhances the resolution, producing a final image with doubled resolution.

### A design strategy for a multifocal metalens

The generation of a high-density multifocal array plays an important role in implementing ISM, particularly in terms of scanning speed and reconstruction quality. In the metalens field, various design methods have been suggested to create multifocal arrays with numerous foci, such as metalens arrays^22,33,34^, utilizing diffraction gratings^2,18,19,35^, and multiplexing phase profiles^36^. However, the metalens array exhibits a tradeoff between the NA and the number of foci. Diffraction grating-based multifocal arrays have limitations in terms of uniformity and the number of foci owing to diffraction angles. Additionally, in the multiplexing phase profiles, the number of available foci is constrained by the size of the metalens.

**Fig. 2:**
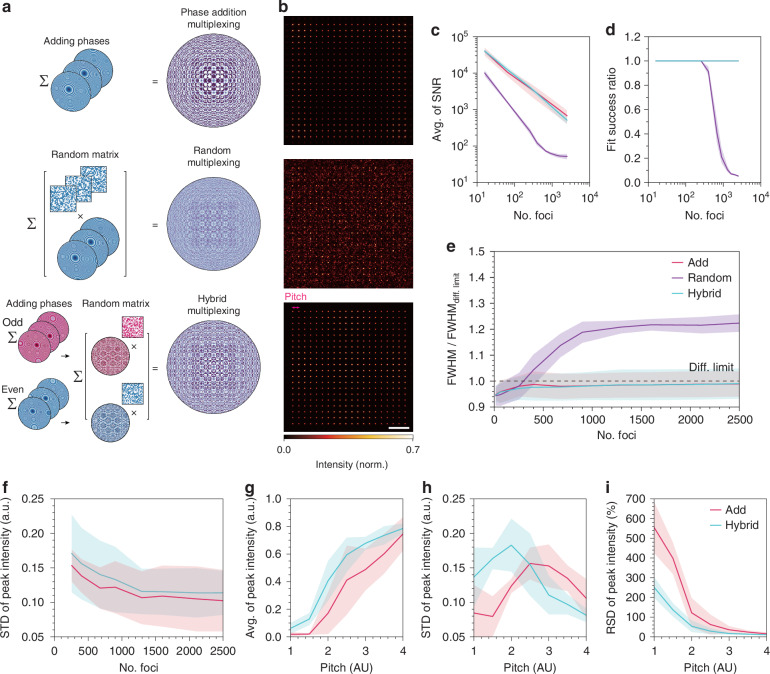
**Characterization and comparison of the conventional and hybrid multiplexing methods for multifocal metalens.** **a** Schematics of conventional multiplexing methods (phase addition and random multiplexing) and the proposed method (hybrid multiplexing). **b** Representative simulated PSFs of multifocal arrays generated using phase addition (top), random multiplexing (middle), and hybrid multiplexing (bottom). The multifocal arrays were designed with parameters of 20 × 20 foci, 3 μm pitch, 0.7 NA at a wavelength of 488 nm. Scale bar: 10 AU. **c**–**e** Comparison of the quality of multifocal arrays in terms of resolution and SNR generated by the three multiplexing methods. The SNR (**c**), fit success ratio (**d**), and estimated FWHM in the simulation divided by the theoretical FWHM (**e**) are plotted with respect to the number of foci. The fit success ratio indicates the number of successful Gaussian fits divided by the total number of foci. **f**–**i** Analysis of the quality of the multifocal arrays in terms of uniformity and intensity. **f** Uniformity of peak intensity in STD with respect to the number of foci. The average peak intensity (**g**), uniformity in STD (**h**), and uniformity in RSD of the multifocal arrays as a function of pitch (**i**). The diameter and NA for the simulation in (**c**–**i**) were 500 μm and 0.7, respectively. Error bars in (**c**–**i**) represent the STD

In this study, we introduced a novel design strategy, hybrid multiplexing which combines two conventional phase multiplexing techniques (phase addition multiplexing^37,38^ and random multiplexing^36,39–41^) to generate high-density multifocal array (Figs. 1b and 2a). The addition of phase profiles is the most common method of creating multiple foci with a single metalens. The integrated phase profile (${\psi }_{{\rm{A}}}$) of a multifocal metalens designed using the phase addition method, which generates a *n* × *n* regular grid of foci, can be expressed as (Fig. 2a)^23^:

1 $${\psi}_{{\rm{A}}}\left(x,y\right)={\rm{arg}}\left[\mathop{\sum }\limits_{i=0}^{n-1}\mathop{\sum }\limits_{j=0}^{n-1}{e}^{i{\varphi }_{{ij}}(x,y)}\right]$$

2 $$\begin{array}{c}{\varphi }_{{ij}}\left(x,y\right)=-\,\frac{2\pi }{\lambda }\left(\sqrt{{\left[x-\left\{{x}_{0}+\left[i-\frac{n-1}{2}\right]s\right\}\right]}^{2}+{\left[y-\left\{{y}_{0}+\left[j-\frac{n-1}{2}\right]s\right\}\right]}^{2}+{f}^{2}}-f\right),\\ {\rm{where}}\,{i},\,{j}\in \left\{0,\,1,\,2,\,\ldots ,\,n-1\right\}\end{array}$$

where $n$, ${\varphi }_{{ij}}$, $f$, $({x}_{0},\,{y}_{0})$, $\lambda$, and $s$ denote the number of foci in each column and row, the phase profile of each individual metalens, focal length, center of the *n* × *n* multifocal array, wavelength of the incident light, and pitch between adjacent foci, respectively. However, the phase addition method has disadvantages when generating closely spaced foci owing to interference (Fig. S2 and Supplementary Notes. 1). Theoretically, creating foci using this method (Eq. (1)) is optically the same as coherently superimposing an individual focus, which causes interference among them (Supplementary Notes. 2). This interference becomes more evident as the focal points are positioned closer together, resulting in artifacts such as undesirable side lobes and an inhomogeneous intensity distribution of the foci (Fig. S3).

Although incoherent foci are less sensitive to interference, obtaining them using a single metalens is challenging. A promising alternative is random multiplexing, which is commonly used to combine multiple holograms (Fig. S3)^26,27^. This method involves multiplying binary random matrices by the phase maps to be integrated, followed by summing the phase maps. The equation for the phase profile of a multifocal metalens (${\psi }_{{\rm{R}}}$) using random multiplexing is as follows^24^:

3 $${\psi }_{{\rm{R}}}\left(x,y\right)=\mathop{\sum }\limits_{i=0}^{n-1}\mathop{\sum }\limits_{j=0}^{n-1}{\varphi }_{{ij}}\left(x,\,y\right)\times {L}_{{ij}}\left(x,\,y\right),{\rm{where}}\,{L}_{{ij}}\left(x,\,y\right)\in \{0,\,1\}$$

4 $$\mathop{\sum }\limits_{i=0}^{n-1}\mathop{\sum }\limits_{j=0}^{n-1}{L}_{{ij}}(x,\,y)=1\,\forall (x,\,y)$$

where $${n}^{2}$$ is the number of multiplexing, and $$L(x,{y})$$ is the set of binary random matrices that satisfy the condition that at each position ($$x,{y})$$, only one matrix ($${L}_{{ij}})$$ for some indices $$i$$ and $$j$$ must have a value of 1, while all other matrices are 0 (Eq. (4)). The sparsity of all random matrices, defined as the fraction of the area where the elements have a value of 1, was set as $${1/n}^{2}$$ of the total area of the metalens. The random multiplexing method was less sensitive to interference than the phase addition method (Fig. S3), thus providing more uniform multifocal arrays. The results from the 1D multifocal array showed that the intensity uniformity of the foci generated by random multiplexing was comparable to that of incoherent foci (Fig. S3). However, this method dramatically degraded the quality of the foci as the multiplexing increases^36^ (Fig. 2b). The random matrix becomes a sparser with an increase in multiplexing, leading to the degradation of the individual phase profiles because this method involves multiplying the random matrices by the phase profile. Indeed, according to optical propagation theory, the peak intensity of a certain focus using random multiplexing is proportional to the square of the metalens area and inversely proportional to the number of multiplexing (Supplementary Notes. 3). Consequently, creating numerous foci using random multiplexing methods is limited in terms of the foci quality.

To address this issue, we introduce a hybrid multiplexing method, aimed at creating high-density homogeneous multifocal arrays for implementing MMISM. We hybridized the conventional multiplexing methods (phase addition and random multiplexing) to leverage their advantages while minimizing their disadvantages. The phase profiles for hybrid multiplexing (${\psi }_{{\rm{H}}}$) can be expressed as:

5 $$\begin{array}{c}\psi \left(x,y\right)={\rm{arg}}\left[\mathop{\sum }\limits_{i+j=0}^{{(n-1)}^{2}}{e}^{i{\varphi }_{{ij}}(x,y)}\right]\\ {\rm{where}}\,\psi \left(x,\,y\right)=\left\{\begin{array}{l}{\psi }_{{\rm{even}}}\left(x,\,y\right)\,{\rm{if}}\,\left(i+j\right)\,{\rm{is}}\;{\rm{even}}\\ {\psi }_{{\rm{odd}}}\left(x,\,y\right)\,{\rm{if}}\,\left(i+j\right)\,{\rm{is}}\;{\rm{odd}}\end{array}\right.,\,i,\,j\in \left\{0,\,1,\,2,\,\ldots ,\,n-1\right\}\end{array}$$

6 $${\psi }_{{\rm{H}}}\left(x,y\right)={\psi }_{{\rm{even}}}\left(x,y\right)\,{L}_{1}\left(x,\,y\right)+\,{\psi }_{{\rm{odd}}}\left(x,y\right)\,{L}_{2}\left(x,\,y\right)$$

where ${\psi }_{{\rm{even}}}$ and ${\psi }_{{\rm{odd}}}$ are the phase maps for generating mutually interleaved multifocal arrays, and ${L}_{1}\left(x,{y}\right)$ and ${L}_{2}\left(x,{y}\right)$ are binary random matrices that satisfy ${L}_{1}\left(x,{y}\right)+\,{L}_{2}\left(x,{y}\right)=1$ for all $\left(x,{y}\right)$. The resulting pitch between adjacent foci was $s/2$ for the hybrid multiplexing method (Eq. (2)). First, we split the regular grid of the multifocal array into two mutually staggered multifocal grids to increase the pitch between adjacent foci, thereby preventing interference (Fig. 1b). Notably, each phase map, termed even and odd phases, is designed using the phase addition method to maintain the quality of the numerous foci (Eq. (5)). Then, the even phase (${\psi }_{{\rm{even}}}$) and the odd phase (${\psi }_{{\rm{odd}}})$ were integrated through random multiplexing to minimize interference between adjacent foci (Eq. (6)). Notably, only two random matrices were used to combine ${\psi }_{{\rm{even}}}$ and ${\psi }_{{\rm{odd}}}$, which prevents the degradation of multifocal array quality (Supplementary Notes. 4). The simulation results clearly showed that the proposed method produced more uniform, denser, and higher-quality foci than the conventional methods when the number of foci and pitch were the same (Fig. 2b).

### Optimization of the multifocal metalens design

We performed wave propagation simulations to optimize the performance of the multifocal metalens (see Methods). The major parameters affecting the quality of the multifocal array include the wavelength, NA, diameter of the metalens, number of foci, and pitch. Since multifocal metalens distribute light across multiple foci, achieving high focusing efficiency is critical factor for fluorescence imaging applications. Generally, increasing NA results in a significant drop in focusing efficiency^42–45^. Moreover, high-NA metalenses require small meta-atom periods, which lead to more frequent fabrication defects and further decrease focusing efficiency. We conducted electromagnetic simulations to investigate the NA dependency of focusing efficiency and determine the optimal NA (Fig. S4). We selected an NA of 0.7 for our multifocal metalens design, as the focusing efficiency suddenly declined beyond this value. Throughout this study, we used a wavelength of 488 nm and an NA of 0.7 unless otherwise stated. For the hybrid multiplexing design, we used multiple spherical metalens phase profiles, each with an NA of 0.7, while applying different lateral shifts to ensure a uniform pitch between foci (Eqs. (2), (5)). Since the multifocal metalens consists of metalens phase profiles with the same NA, we defined its NA based on that of the individual metalens. The wave propagation simulations were conducted with various pitches (1–4 airy units, AU) and the number of foci (16–2500) at a fixed diameter of 500 μm to examine the effects of these parameters on the quality and uniformity of the multifocal array (Figs. S5 and S6). We evaluated the quality, uniformity, and resolution by measuring the average peak intensity (or SNR), standard deviation (STD) (or relative standard deviation, RSD), and FWHM, respectively.

Because integrating multiple phase profiles reduces the weight of each individual phase profile, increasing the number of foci inevitably leads to degradation of the SNR, regardless of the pitch (Fig. 2c and Fig. S6). The primary noise sources that degrade focal spot quality are interference and diffraction, which undesirably redistribute energy outside the focal spots. To quantify the degradation in focal quality caused by these noise sources, we evaluated the SNR with respect to the number of foci (Fig. 2c). As mentioned earlier, the random multiplexing method was more susceptible to degradation of the beam quality as the number of foci increased (Fig. 2c). We also evaluated the FWHM of all the simulated foci using Gaussian fitting for comparison with the theoretical diffraction-limited resolution. The fitting results revealed that the foci generated using the phase addition and hybrid multiplexing methods were well fitted to a Gaussian function, and the obtained resolutions were comparable to the theoretical FWHM at the diffraction limit^46,47^. However, for random multiplexing, the curve-fitting success ratio suddenly decreased when the number of foci exceeded 300 (Fig. 2d and Fig. S7). The estimated resolutions from the successfully fitted foci in the simulation were up to 20% greater than the diffraction limit (Fig. 2e). Because of the difficulty of Gaussian fitting for random multiplexing, particularly with numerous foci, we focused on comparing the phase addition and hybrid multiplexing methods throughout the optimization studies.

Contrary to the SNR, the uniformity of the foci generally improved as the number of foci increased, showing a plateau after ~1500 foci (Fig. 2f). The pitch also significantly influenced both the average intensity and uniformity (Fig. 2g, h). The average peak intensity increased with a larger pitch, which was likely due to the reduced interference between adjacent foci. Notably, the foci generated using the hybrid multiplexing method exhibited higher intensities than those generated using the phase addition method. Although the STD of the foci did not follow a consistent trend with respect to the pitch, the RSD, which reflected both the uniformity and brightness of the multifocal array, gradually improved with increasing pitch (Fig. 2i). In summary, increasing the number of foci has advantages in terms of uniformity and FOV for ISM but reduces the SNR. Although decreasing the pitch is required to enhance the ISM scanning speed by reducing the number of frames required for reconstruction, it has disadvantages in terms of uniformity, brightness of foci, and the FOV of the ISM. Considering these tradeoffs, we selected the parameters of 40 × 40 foci where the uniformity reached a plateau (Fig. 2f) and a pitch of 3.5 AU for fabrication. Although the pitch value is not optimal for the RSD (Fig. 2i), it provides a good balance when considering the FOV of the ISM. This configuration still offers better performance than the phase addition method (Fig. S8).

The lens diameter and NA are also critical factors for improving beam quality. Larger diameters collect more light, enhancing the SNR (Supplementary Notes. 2–4). Furthermore, a larger NA reduces the FWHM of the foci, suppresses the interference between closely spaced focal points, and improves the SNR. To investigate the dependency on NA and diameter, we carried out wave propagation simulations with various NA (0.3–0.7) and diameter (300–1000 μm), while keeping the number of foci (40 × 40) fixed at the previously optimized values (Figs. S9 and S10). It is important to note that we used a pitch of 3 μm in physical units, corresponding to 3.5 AU at 488 nm with a NA of 0.7. This is because the use of the dimensionless optical unit AU can result in a varying physical pitch depending on the NA, even though the pitch in the dimensionless optical unit remains constant. Such variability in the physical pitch is not desirable for this simulation, which aims to observe only the effect of the NA on the beam quality. As expected, the beam quality was proportional to both the diameter and NA at a fixed pitch and number of foci (Fig. S11). We selected a diameter of 1000 μm and an NA of 0.7 to fabricate multifocal metalens for constructing ISM. With these parameters, we anticipate achieving a 120 μm FOV, corresponding to an angular FOV of ~13.5°.

### Fabrication and characterization of the multifocal metalens

**Fig. 3:**
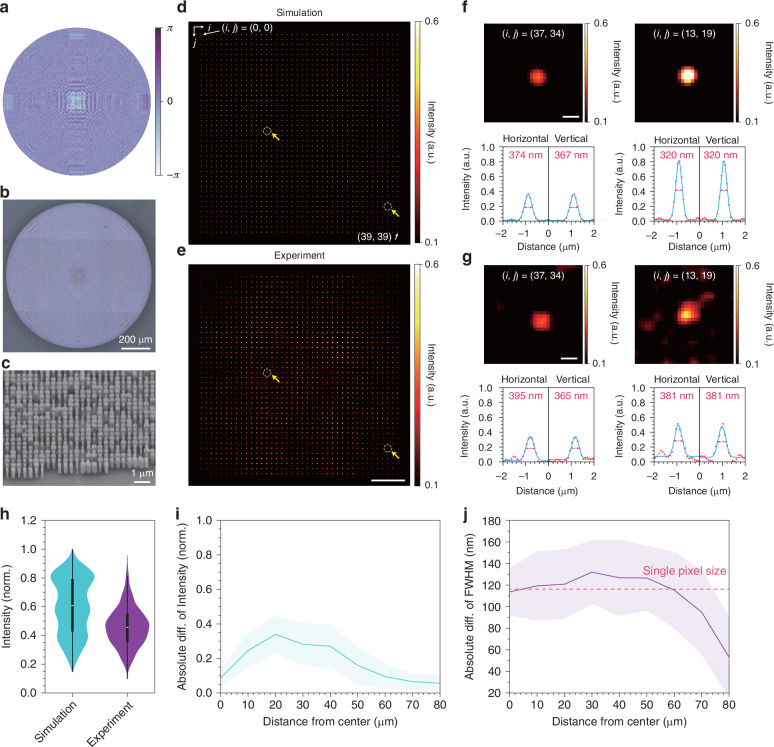
**Demonstration of the fabricated multifocal metalens.** **a** Phase profile of the multifocal metalens generating 40 × 40 foci with a 3 μm pitch and 0.7 NA under 488 nm light illumination. **b** Optical microscopy image of the fabricated multifocal metalens. **c** Scanning electron microscopy (SEM) image of the SiN meta-atoms on the fabricated multifocal metalens. **d** Numerically simulated PSF of the designed multifocal metalens. **e** Experimentally obtained PSF of the fabricated multifocal metalens. To ensure accurate comparison with the simulation, bilinear interpolation was applied to the experimentally acquired PSF image to match the pixel size with the simulation data. Scale bar: 20 μm. **f** Simulated PSF images (top) cropped from (**d**) as indicated by the yellow arrows and the corresponding horizontal and vertical intensity profiles (bottom). Scale bar: 400 nm. **g** Experimentally obtained PSF images (top) cropped from (**e**) as indicated by the yellow arrows and the corresponding horizontal and vertical intensity profiles (bottom). Scale bar: 400 nm. **h**–**j** Quantitative analysis of the multifocal array PSF obtained from simulations and experiments. **h** Normalized average peak intensity of the multifocal array. The thick and thin lines represent the interquartile range (IQR) and 1.5 IQR, respectively. The white line in the center denotes the median value. The violin plot shows the entire range of data points. **i** Absolute intensity difference between the simulated and measured multifocal array images with respect to the distance from the center. **j** Absolute difference in FWHM with respect to the distance from the center. Error bars in (**h**–**j**) represent the STD

To create a meta-atom library, we performed rigorous coupled-wave analysis (RCWA) based on the measured refractive index of SiN at 488 nm (Fig. S12). Subsequently, multifocal metalens was fabricated using electron beam lithography (EBL) to pattern the desired meta-atom structure, followed by lift-off and etching processes (Fig. 3a–c; see Methods). The performance of the multifocal metalens was experimentally validated and compared with the simulation results (Fig. 3d, e). Remarkably, the position of each focus in the experimentally obtained multifocal array agrees well with the simulation results (Fig. S13). For a more rigorous analysis, min-max normalization was applied to the multifocal array image, and all foci were fitted with a Gaussian function to evaluate the resolution and peak intensity (Fig. 3f, g). The experimentally measured average peak intensity was ~14% lower than that of the predicted value (Fig. 3h), with the greatest discrepancy occurring at distances of 20–30 μm from the center of the multifocal array (Fig. 3i). However, the size of each point spread function (PSF) exhibited trends that differed from the distribution of the peak intensity. The deviations in PSF size from the simulated values were comparable to the single-pixel size (~110 nm) of our system up to a distance of 60 μm from the center, after which the difference diminished toward the outermost region (~80 μm). We expect that the deviations in the intensity and resolution distributions arise from fabrication defects, the quality of the light source, the large pixel size of our camera, and the slight tilt of the metalens. Despite the deviations from simulation, the performance of our multifocal metalens, which generates 40 × 40 foci with 3 μm pitch and 0.7 NA, is better than the conventional optical elements, such as MLAs.

### Design and fabrication of polarization multifocal metalens

As previously described, although reducing the pitch has the advantage of decreasing the number of frames required for ISM reconstruction, it is constrained by the interference among coherently generated foci. In general, generating incoherent and high-NA foci is not feasible using conventional diffractive optical elements^48^, spatial light modulators^49^, or even metalenses designed for unpolarized light. However, polarization metalenses based on the Pancharatnam-Berry (PB) phase^50,51^ can simultaneously modulate two orthogonally polarized lights that do not interfere with each other, allowing for the production of high-NA foci with small pitch. We assume that a narrower pitch can be achieved by independently modulating right-handed circularly polarized (RCP) and left-handed circularly polarized (LCP) light, which is a commonly used method to create multifunctional or switchable metalenses^52–54^.

Here, we propose a polarization hybrid multiplexing method that applies the PB phase-based independent control of RCP and LCP light to a hybrid multiplexing approach (Fig. S14). The key is to create mutually staggered LCP and RCP multifocal arrays, ensuring that each focus is surrounded by other foci with orthogonal polarization to prevent interference. Orthogonally polarized multifocal arrays were designed using a hybrid multiplexing method according to the following equations:

7 $$\begin{array}{c}\psi \left(x,y\right)={\rm{arg }}\left[\mathop{\sum }\limits_{i=0}^{n-1}\mathop{\sum }\limits_{j=0}^{n-1}{e}^{i{\varphi }_{{ij}}(x,y)}\right]\\ {\rm{where}}\,\psi (x,\,y)=\left\{\begin{array}{ll}{{\psi }_{{\rm{LCP}},{\rm{even}}}\left(x,\,y\right)\,{\rm{if}}\,i,\,j\,{\rm{are}}\; {\rm{even}}}\\{{\psi }_{{\rm{LCP}},{\rm{odd}}}\left(x,\,y\right)\,{\rm{if}}\,i,\,j\,{\rm{are}}\;{\rm{odd}}}\\{{\psi}_{{\rm{RCP}},{\rm{even}}}\left(x,\,y\right)\,{\rm{if}}\,i\,{\rm{is}}\;{\rm{odd}}\;{\rm{and}}\,j\,{\rm{is}}\;{\rm{even}}}\\{{\psi}_{{\rm{RCP}},{\rm{odd}}}\left(x,\,y\right)\,{\rm{if}}\,i\,{\rm{is}}\;{\rm{even}}\;{\rm{and}}\,\,j\,{\rm{is}}\;{\rm{odd}}}\end{array}\right.,\,\quad{i},\,\,j\in \left\{0,\,1,\,2,\,\ldots ,\,n-1\right\}\end{array}$$

8 $${\psi }_{{\rm{LCP}}}\left(x,y\right)={\psi }_{{\rm{LCP}},{\rm{even}}}\left(x,y\right)\,{L}_{1}\left(x,\,y\right)+\,{\psi }_{{\rm{LCP}},{\rm{odd}}}\left(x,y\right)\,{L}_{2}\left(x,\,y\right)$$

9 $${\psi }_{{\rm{RCP}}}\left(x,y\right)={\psi }_{{\rm{RCP}},{\rm{even}}}\left(x,y\right)\,{L}_{1}\left(x,\,y\right)+\,{\psi }_{{\rm{RCP}},{\rm{odd}}}\left(x,y\right)\,{L}_{2}\left(x,\,y\right)$$

where ${\psi }_{{\rm{LCP}}}$ and ${\psi }_{{\rm{RCP}}}$ are the target phase profiles for generating mutually interleaved multifocal arrays for each circularly polarized light. The subscripts ‘even’ and ‘odd’ denote the corresponding even and odd phases for each polarization. The resulting pitch between adjacent foci was $s/2$ for the polarization hybrid multiplexing method (Eq. (2)). To investigate the performance of this method, wave propagation simulations were conducted for various numbers of foci (25–1600) and pitches (1–4 AU) at a fixed diameter of 500 μm (Figs. S15 and S16). As expected, the multifocal arrays produced using the polarization hybrid multiplexing method exhibited better uniformity than those produced using the phase addition or hybrid multiplexing methods, even at small pitches ( <3 AU) (Fig. S17).

As a proof of principle, we fabricated a multifocal metalens using a polarization hybrid multiplexing method to create denser and more homogeneous multifocal arrays. To independently control the RCP and LCP, we selected hydrogenated amorphous silicon (a-Si:H)^55^, which is known for its high refractive index, and performed RCWA simulations to create a meta-atom library (Fig. S18; see Methods). We patterned a phase map for a multifocal metalens that creates 12 × 12 foci using EBL on 650 nm thick a-Si:H with a diameter of 100 μm and a pitch of 2 AU, designed to operate at a wavelength of 633 nm. When either RCP or LCP light was incident on the multifocal metalens, mutually interleaved multifocal arrays were formed at the focal plane, depending on the polarization state. Furthermore, the metalens produced a complete grid of multifocal array when illuminated with unpolarized light (Fig. S19). According to the simulation, the normal hybrid multiplexing method exhibited a less uniform intensity distribution across the multifocal array than the polarization hybrid multiplexing method under the same conditions (Fig. S19). Although some fluorophores exhibit circular dichroism with distinct absorption responses to LCP and RCP light, the absorption difference is negligible compared to their molar extinction coefficient (<0.5%)^56^. Conversely, circular dichroism can potentially be exploited for chiral MMISM imaging by utilizing multifocal metalenses with polarization hybrid multiplexing design and modified focal spot arrangements^57^.

### Multifocal metalens-based ISM

ISM is a super-resolution imaging technique that achieves diffraction-limited resolution through pixel reassignment^2–4,25^. Theoretically, the resolution of ISM can be improved by a factor of $\sqrt{2}$ through pixel reassignment and ultimately doubled with deconvolution compared to the diffraction-limit^2,8^. To enable pixel reassignment, it is essential to use an array detector with pixels smaller than the PSF to ensure that the PSF is distributed across multiple pixels. Each pixel in the array detector acts as a single detector with a pixel-sized pinhole. One simple way to implement the ISM is to use multifocal array illumination and a camera (sCMOS or CCD) for array detection.

**Fig. 4:**
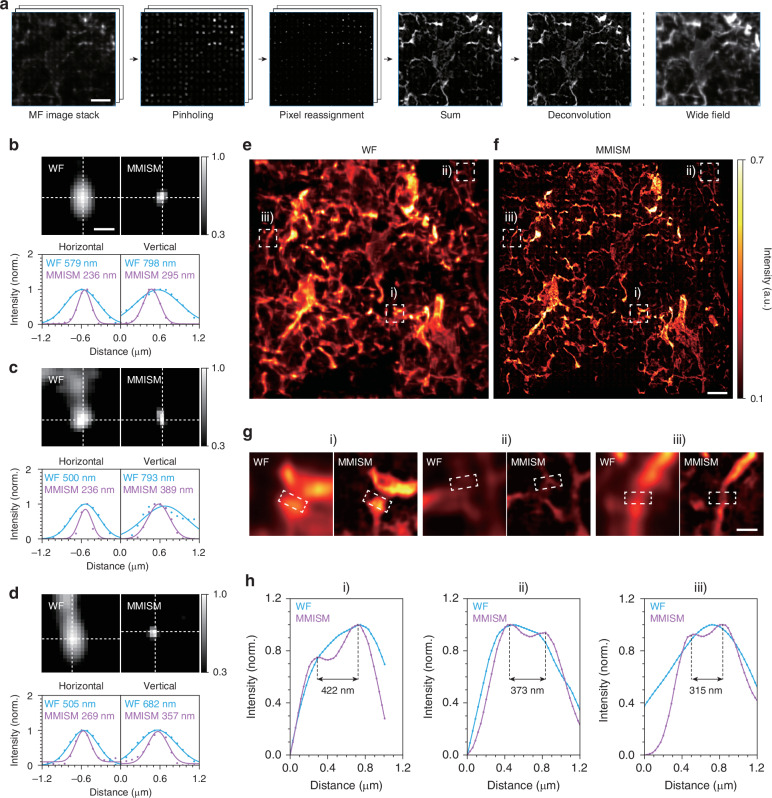
**MMISM imaging of human forebrain organoids and microbeads.** **a** The image processing pipeline for MMISM reconstruction. The reconstruction process involves acquiring multifocal image stack, applying macro pinholes, performing pixel reassignment, summation, and deconvolution. Scale bar: 10 μm. **b**–**d** WF (top left) and MMISM (top right) images of 0.03 μm microbeads with corresponding horizontal (bottom left) and vertical (bottom right) intensity profiles. The white dashed lines represent the directions of the plotted intensity profiles. The FWHM resolution measured by MMISM (~240–390 nm) is approximately twice as high as that of WF (~500–800 nm). The WF images are upsampled by a factor of 2 to align particle locations with MMISM images. Scale bar: 500 nm. **e**, **f** WF (**e**) and MMISM (**f**) images of a sectioned organoid sample immunohistochemically stained with MAP2 neuronal marker. The MMISM image shows clearer and more distinct fiber structures compared to the WF image. The WF image is upsampled by a factor of 2 to align cellular structures with the MMISM image. Scale bar: 10 μm. **g** Magnified images (i–iii) correspond to the dashed boxes in (**e**, **f**). Scale bar: 2 μm. **h** Intensity profiles from the selected regions (i–iii) as indicated by the dashed lines in (**g**). The MMISM successfully resolved fine features separated by ~300–400 nm, whereas the WF was unable to distinguish between these structures

In this study, we developed a customized fluorescent ISM equipped with the fabricated multifocal metalens (40 × 40 foci and 3 μm pitch) for multifocal array illumination, covering a FOV of 120 μm (Fig. 1c and Fig. S20; see Methods). The multifocal array PSF was relayed to the sample plane without magnification using two ×20 objective lenses (NA 0.5). The objective lens NA was chosen by considering the tradeoff between resolution and scattering in turbid organoid tissue, as high-NA lenses suffer from increased scattering noise in turbid media^58–60^. We scanned the multifocal array PSF laterally and vertically in single-pixel steps (172.5 nm) using a motorized stage to ensure seamless imaging of the entire FOV for ISM reconstruction. The number of scans in each direction was determined using the following equation:

10 $${NS}=\left[\frac{s}{p}\right]$$

where ${NS}$ is the number of scans in a single direction, $p$ is the pixel size, and the square bracket denotes the flooring function, for example, $\left[x\right]$ returns the greatest integer less than or equal to $x$. In our setup, a total of 289 frames, corresponding to $N{S}^{2}$, were required for a single ISM reconstruction. Here, ${NS}$ was 17 frames obtained using a pitch of 3 μm (3.5 AU@488 nm) and a pixel size of 172.5 nm (Eq. (10)). Notably, as mentioned earlier, reducing the pitch size by half can decrease the number of required frames by a factor of four (Eq. (10)), thereby increasing the imaging speed. After multifocal scanning, ISM reconstruction was applied to the acquired image stacks (Fig. 4a; see Methods)^5,8,61^.

We experimentally demonstrated the MMISM using 0.03 μm fluorescent beads and 40 μm-thick organoid tissues. To characterize optical resolution, we first recorded image stacks of fluorescent beads under multifocal illumination. The FWHM values for the fluorescent beads obtained using a WF microscope were ~590–720 nm, which is slightly larger than the theoretical FWHM of 530–550 nm. The deviations of the measured resolution from the theoretical value are presumably caused by the large single-pixel size (~173 nm) and system aberrations. Notably, the resolution nearly doubled with MMISM reconstruction, achieving an FWHM of ~330–370 nm (Fig. 4b–d and Figs. S21–23). We observed larger average vertical resolution values compared to horizontal resolution for both WF (591 nm horizontal and 717 nm vertical) and MMISM (327 nm horizontal and 373 nm vertical) (Figs. S21–23), presumably due to system aberrations and imperfections in metalens fabrications.

Next, we further demonstrated super-resolution imaging and confocal detection using MMISM with thick biological samples. Precise recording of complex neuronal structures is essential for elucidating neuronal functions and connections, as well as studying brain dysfunction and neurodegenerative diseases. In contrast to bead samples, the confocal detection capability of MMISM, which is not available in WF imaging, becomes even more crucial for thick tissue imaging, as scattering disturbs the identification of features (Fig. S1). We obtained image stacks under multifocal illumination and performed ISM reconstruction on 40 μm-thick forebrain organoid sections stained with MAP2 and pTau antibodies, which labeled microtubule-related proteins and displayed neuronal structures. Typically, scattering becomes significant beyond a thickness threshold of approximately 10–20 μm^62,63^. WF imaging of tissues exceeding this thickness suffers from severe scattering^64^, requiring advanced optical sectioning techniques, such as confocal microscopy and digital pinholing, to obtain clear images without scattering noise. Compared with the WF images, the super-resolution MMISM images with confocal detection showed clearer and more distinct structural features (Fig. 4e–g and Fig. S24). Notably, Fig. S24a clearly demonstrates the out-of-focus signal rejection performance of digital pinholing, where the blurred features observed in WF imaging are removed in MMISM. As shown in the representative intensity profiles (Fig. 4h), the neuronal structures separated by ~300–400 nm were well-resolved using MMISM, whereas WF failed to resolve them. These results demonstrate the performance of the proposed MMISM equipped with the multifocal metalens designed using the novel hybrid multiplexing method.

## Discussion

In conclusion, we developed a novel method called hybrid multiplexing for designing multifocal metalens that create dense and uniform multifocal arrays. Our simulation results revealed that the proposed method offers a greater number of foci, smaller pitch, and improved uniformity compared to conventional multiplexing techniques. In addition, adopting independent polarization control allows for a reduction in pitch while maintaining the foci uniformity. As a proof of concept, we fabricated a multifocal metalens using a hybrid multiplexing method and constructed an MMISM system. The feasibility of MMISM was demonstrated in both microbead samples and sectioned organoid samples. The experimental results indicated that the MMISM images achieved approximately double the resolution of the WF images. Moreover, the neuronal structures exhibited clearer and more distinct features, attributed to the sub-diffraction-limited resolution and optical sectioning effects of the MMISM.

In this study, we selected a pitch of 3.5 AU for the multifocal metalens used in MMISM to obtain a large FOV, although hybrid multiplexing provides uniform multifocal arrays even at smaller pitches (<3 AU). The FOV of the MMISM can be increased by increasing the number of foci; however, this approach is limited by the degradation of the beam quality. This limitation can be overcome by employing larger metalens that have more multiplexing capabilities, thereby providing a wider FOV (Figs. S10 and S11). Typically, diameters of only 1–2 mm are available at laboratory scale. To overcome this limitation, the mass production of large metalenses has been actively studied^65–67^. Notably, the mass production of centimeter-scale metalenses using nanoimprint lithography has been reported^68^. These developments suggest the potential for scalable production of large multifocal metalenses, resulting in faster and broader MMISM capabilities.

Another potential advancement is achieving multispectral capability for obtaining spectral information, a crucial aspect of bioimaging^69^. For multispectral applications, focusing different wavelengths on the same focal plane is essential. The simplest approach involves random multiplexing^70^, which combines wavelength-specific phase maps using random matrices. While effective for a few distinct wavelengths with sufficiently large separations, this method consumes multiplexing capacity. A dual-mode metalens that independently modulates the phase for orthogonal polarization at different wavelengths could also be a solution, though it is limited to only two wavelengths available^71^. Alternatively, incorporating an achromatic metalens design into our multifocal metalens could be a breakthrough^34,72–74^. While integrating achromatic design presents significant challenges, its successful implementation could allow arbitrary wavelength selection for multispectral imaging applications.

In our study, one aspect that could be further improved is integrating modulation and detection paths into a single system, thereby further simplifying the setup. Fluorescence microscopy inevitably requires separation of light modulation and detection paths due to differences in excitation and emission wavelengths, which is also valid for refractive optics. The recently reported nonlocal Huygens’ metalens presents a promising solution to this problem, as it exhibits wavelength selectivity, modulating only specific narrowband light while leaving others unmodulated^75^. This capability could potentially enable shared light modulation and detection paths through a single multifocal metalens, which may facilitate the elimination of remote focusing systems for MMISM. In addition, leveraging this property could potentially lead to the design of more compact and efficient optical systems, enhancing imaging performance while reducing complexity.

Compared to other metalens-based super-resolution microscopy, MMISM offers several benefits in terms of penetration depth for biological imaging. Previously reported metalens-based super-resolution microscopy can be broadly classified into two categories: (i) near-field imaging^69,76–80^ and ii) sub-diffraction PSF forming^69,81–83^. Near-field-based super-resolution metalenses, such as hyperlenses and superlenses, inherently require direct contact between the sample and metalens due to the restricted propagation of near-field components smaller than the wavelength. While these metalenses offer dramatic resolution enhancements, their applicability to deep tissue imaging is limited. Generating a sub-diffraction-limited PSF with metalens, such as a super oscillatory lens, enables the far-field implementation of super-resolution microscopy. However, this method often relies on computationally burdensome and complex optimization algorithms for metalens design and suffers from slow imaging speed due to the raster scanning nature, as it operates with only a single focus. While MMISM also has some advantages, such as far-field nature and fast scanning via multifocal illumination, its most notable feature is its deep tissue imaging capability, distinguishing it from previous super-resolution metalenses. Generally, WF microscopy suffers from scattering in tissues thicker than 10–20 μm^62–64^. In this study, MMISM successfully achieved super-resolution imaging in 40 μm-thick brain organoid tissue by means of digital pinholing algorithms. As far as we know, this is the first implementation of metalens-based super-resolution imaging in thick biological tissues rather than in cultured cells. However, the maximum penetration depth of MMISM could be smaller than that of conventional confocal microscopy (<100–500 μm)^84–87^. This limitation arises because MMISM performance relies on the peak detection algorithm, which determines the exact digital pinhole position, but it works badly in highly scattering environments. Designing a multifocal metalens compatible with a multiphoton laser could be an effective solution to mitigate this issue^88,89^, as the low scattering nature of near-infrared light in tissue improves penetration depth. Enhancing deep tissue imaging capabilities could further enable large-volume 3D super-resolution imaging in thick tissues, potentially applying it to 3D structural analyses, such as neuronal morphology reconstruction.

A hybrid multiplexing strategy can be used to combine arbitrary phase maps for general purposes. Therefore, the proposed design approach can be applied not only to multifocal metalenses but also to various types of multifunctional metalens designs. This versatility has the advantage of integrating multiple optical elements into a single thin metalens, leading to a simpler and more compact imaging system. Although metalenses may not fully replace conventional optical elements for general purposes, we anticipate that they can serve as alternatives to optical components specialized for specific applications, such as MMISM. We believe that these achievements open up new possibilities for metalens-based imaging systems, not only for MMISM, but also for promising advanced optical microscopy systems.

## Materials and methods

### Numerical simulations

TORCWA^90^, a Python library for RCWA electromagnetic simulation, was used to create the meta-atom libraries. The complex refractive index of SiN was used to simulate the cylindrical structure of meta-atoms with unpolarized light at a wavelength of 488 nm. For polarization-dependent rectangular meta-atoms, the complex index of a-Si:H was used. Silicon dioxide (SiO_2_) was used as the substrate. The heights of the meta-atoms were set to 750 and 650 nm for SiN and a-Si:H, respectively. All complex refractive index data were measured using ellipsometry.

The phase profile of the multifocal metalens was calculated using the CuPy Python library to accelerate the matrix operation with a GeForce RTX 4090 (NVIDIA) GPU. The polarization-independent multifocal metalens operating at 488 nm was optimized under various conditions, and a NA of 0.7, pitch of 3.5 AU, diameter of 1 mm, and 40 × 40 foci were selected for fabrication. The airy unit AU can be expressed as ${\rm{AU}}=\frac{1.22\lambda }{{\rm{NA}}}$. On the other hand, the polarization-dependent multifocal metalens operating at 633 nm was designed to have an NA of 0.7, pitch of 2 AU, diameter of 100 µm, and 12 × 12 foci. Rayleigh–Sommerfeld propagation was used to numerically simulate the PSF of the designed multifocal metalens. The intensity for coherently superimposed fields was calculated as $I={\left|\mathop{\sum}\nolimits_{k=1}^{n}{u}_{k}\right|}^{2}$, whereas for incoherently superimposed fields, it was determined by $I=\mathop{\sum }\nolimits_{k=1}^{n}{\left|{u}_{k}\right|}^{2}$, where $I$ represents the resulting intensity, and ${u}_{k}$ denotes the individual fields being interfered with. The equation for incoherent superposition was employed to simulate the performance of a multifocal metalens designed using the polarization hybrid multiplexing method.

### Metalens fabrication

A 750 nm thick SiN was deposited on a SiO_2_ substrate using plasma-enhanced chemical vapor deposition (Oxford, PlasmaPro 100 Cobra) to fabricate a polarization-independent multifocal metalens. Next, a 200 nm thick layer of a positive photoresist (AR-P 6200.09, Allresist) was spin-coated onto the SiN film at 4000 rpm. Then, 100 μL of ESPACER (RESONAC, 300Z) was spin-coated at 2000 rpm for 30 s to prevent charging. The circular meta-atom design was patterned onto the positive photoresist using EBL (NanoBeam, nB5; Dose: 3.75 C m^−2^). The exposed positive photoresist was developed in a 1:3 methyl isobutyl ketone/isopropyl alcohol solution for 690 s. Subsequently, a 40 nm thick chromium (Cr) layer was deposited using an electron beam evaporator (ULVAC, Ei-5k). The unexposed photoresist was removed through the lift-off process using acetone at room temperature (RT) for 2 h, leaving the Cr layer intact as a hard mask. The patterning was completed using inductively coupled plasma etching (ICP; STS, multiplex ICP) with SF_6_ (15 sccm) and C_4_H_8_ gas (40 sccm) for 610 s. Subsequently, the Cr hard mask was removed using a Cr etchant (TRANSENE, CE-905N) for 5 min. To fabricate the polarization-dependent multifocal metalens, the overall process was the same, except that the rectangular meta-atoms were patterned with a 650 nm thick a-Si:H film.

### Optical characterization

The foci of multifocal metalens were characterized using an optical microscope. The 450 nm (Thorlabs, CPS450) and 635 nm (Thorlabs, CPS635) lasers were used as light sources. The beam diameter was adjusted using an iris to match the metalens aperture. The PSFs of the metalens were recorded using a magnification system consisting of a ×100 objective lens (Olympus, 0.9 NA), tube lens (Thorlabs, TTL180-A), and sCMOS camera (Alvium, 1800 U-235). The pixel size of the system is 58.6 nm.

### MMISM setup and data acquisition

The MMISM imaging was performed using a custom-built system (Fig. S20). A 488 nm laser (Coherent OBIS, 488 nm LX 150 mW) was used as an excitation source, and the beam diameter was adjusted to 1 mm using a beam expander and iris. For multifocal illumination, the PSF of the multifocal metalens was relayed onto a sample plane using two identical ×20 objective lenses (Olympus, 0.50 NA) without magnification. The imaging system consisted of a ×20 objective lens, dichromic mirror (Edmund Optics, 67-080), bandpass filter (Edmund Optics, 67-030), tube lens (Thorlabs, TTL180-A), and an sCMOS camera (Thorlabs, CS895MU) to collect the fluorescent signals. The system magnification was ×20, and the pixel size was set to be 172.5 nm. A 3-axis motorized stage (Thorlabs, MT3/M-Z9) was controlled using three DC servo motor controllers (Thorlabs, KDC101) synchronized with the sCMOS camera via a trigger I/O breakout board (Thorlabs, TSI-IOBOB2). Before data acquisition, the rotation angle of the multifocal metalens was aligned parallel to the camera pixels using a rotation mount (Thorlabs, CRM1PT/M). MMISM data were obtained by scanning the motorized stage in both the horizontal and vertical directions with a step size of 172.5 nm under the multifocal illumination. A total of 289 frames (17 × 17) were acquired to seamlessly record the full 120 μm of FOV.

### MMISM reconstruction

The open-source Python code (https://github.com/openUC2/UC2_ISM_CODE)^61^ for ISM reconstruction was used to implement the MMISM reconstruction. The MMISM reconstruction procedures include pinholing, pixel reassignment, image summation, and deconvolution. A Gaussian function was optionally multiplied by a subset image of each individual focus as a macro pinhole to reject out-of-focus light. A macro pinhole size of 770 nm in STD was empirically determined and used^8,11^. After pinholing, pixel reassignment was performed, involving local contraction of the pinholed images and their reassignment into a 2× upsampled image. The pixel-reassigned images were then overlaid and integrated into a single image with a resolution improved by a factor of $\sqrt{2}$. Then, the image was deconvolved using a Gaussian kernel to achieve doubled resolution. The STD of the Gaussian kernel was determined as follows^8^:

11 $${\sigma }_{{\rm{eff}}}^{2}={{m}^{2}\sigma }_{{\rm{em}}}^{2}+{{\left(1-m\right)}^{2}\sigma }_{{\rm{ex}}}^{2}$$

12 $$m=\frac{{\sigma }_{{\rm{ex}}}^{2}}{{\sigma }_{{\rm{ex}}}^{2}+{\sigma }_{{\rm{em}}}^{2}}$$

where the ${\sigma }_{{\rm{eff}}}$, ${\sigma }_{{\rm{ex}}}$, and ${\sigma }_{{\rm{em}}}$ represent STDs of effective, excitation, and emission PSFs, respectively, and $m$ denotes the scaling factor. Practically, a scaling factor of 0.5 is widely adopted (Eqs. (11), (12))^8,91,92^. Wavelengths of 488 and 520 nm were used to determine the sizes of excitation and emission PSFs, respectively.

### Generation of forebrain organoids from iPSCs

Forebrain organoids were derived from human induced pluripotent stem cells (h-iPSCs). The h-iPSCs were initially cultured on Matrigel human embryonic stem cells (hESC)-qualified matrix-coated plates (Corning, 354277) in mTeSR Plus (Stemcell Technologies, ST100-0276). To create embryoid bodies (EBs), h-iPSCs were detached using ReLeSR, and the resulting colonies were dissociated into single cells in AggreWell EB formation medium (Stemcell Technologies, ST05893) with the addition of the ROCK inhibitor Y-27632 (Stemcell Technologies, ST72304). On day 0, 1.5 × 10^6^ cells were loaded into each well of an AggreWell800 24-well plate (Stemcell Technologies, 34811), and the following day, the medium was replaced with EB formation medium without Y-27632. From day 2–5, the medium was changed daily using Dulbecco’s modified eagle medium/nutrient mixture F-12 (DMEM/F-12) with GlutaMAX (Gibco, 10565-018), 20% KnockOut serum replacement (Gibco, A3181501), 1% minimum essential medium (MEM) non-essential amino acid solution (Gibco, 11140050), 0.1 mM 2-mercaptoethanol (Gibco, 21985023), 100 U mL^−1^ penicillin, and 100 μg mL^−1^ streptomycin (Merck, P4333). SMAD inhibitors, including dorsomorphin (10 μM; Merck, P5499) and SB-431542 (10 μM; TOCRIS, 1614), were additionally added. On day 6, 96 EBs were collected, and the following day, each EB was placed in a separate well of a 96-well ultra-low-attachment microplate (Corning, 7007). The organoids were maintained in neural medium containing Neurobasal-A Medium (Gibco, 10888-022), B-27 supplement minus vitamin A (Gibco, 12587010), 100 U mL^−1^ penicillin, 100 μg mL^−1^ streptomycin, GlutaMAX (Gibco, 35050-061), and 0.5% (v/v) Matrigel basement membrane matrix (Corning, 354234). From day 6 to 15, the medium was replaced daily, supplemented with 20 ng mL^−1^ epidermal growth factor (EGF; Peprotech, AF-100-15-500 μg) and 20 ng mL^−1^ fibroblast growth factor basic (bFGF; R&D Systems, 100-18B). Between days 16 and 24, the medium was changed every other day using the same composition. From day 25 to 42, the medium was changed every other day, but EGF and bFGF were replaced with 20 ng mL^−1^ brain-derived neurotrophic factor (BDNF; Peprotech, 450-02) and 20 ng mL^−1^ neurotrophin-3 (NT-3; Peprotech, 450-03). From Day 43 onwards, the medium was refreshed every four days using neural medium without growth factors.

### Immunohistochemistry

Organoids were rinsed with phosphate-buffered saline (PBS; Thermo Fisher Scientific, 14190235), fixed in 4% paraformaldehyde (PFA) at 4 °C for overnight, and then washed again with PBS. They were dehydrated in 30% sucrose at 4 °C for 72 h, transferred to a cryomold, and frozen in FSC 22 compound (Leica, 3801480). Organoids were cryosectioned, washed with PBS, and permeabilized with 0.3% Triton X100 (Merck, X100) in PBS for 30 min at RT. Blocking was performed by incubating the slices in 5% normal horse serum (Sigma-Aldrich, H0146) in PBS for 1 h at RT. Primary antibodies were diluted in the blocking solution and incubated with the slices at 4 °C overnight. Fluorophore-conjugated secondary antibodies were diluted 1:500 in 3% bovine serum albumin in PBS and incubated for 1 h at RT. After washing, sections were mounted onto slides. The antibodies used were against pTau: Thr181 (1:500; Invitrogen, MN1050) and MAP2 (1:500; Abcam, ab254143).

## Supplementary information

Supplementary Information

## Data Availability

The data supporting the findings of this study are available from the corresponding authors upon reasonable request.

## Code availability

The code used in this study is available from the corresponding author upon reasonable request.
