# Supplementary material for: Image scanning microscopy based on multifocal metalens for sub-diffraction-limited imaging of brain organoids
Source: Light Sci Appl. 2025 Oct 13;14:367. doi: 10.1038/s41377-025-01900-3 (PMC12518604; doi:10.1038/s41377-025-01900-3)
Supplement: Supplementary file 1 — Supplementary Information [file 41377_2025_1900_MOESM1_ESM.docx]

**Supplementary Information for**

Image scanning microscopy based on multifocal metalens for sub-diffraction-limited imaging of brain organoids

Yongjae Jo^1, #^, Hyemi Park^1, 2, #^, Seho Lee^1, 2^, Hyeyoung Yoon^2, 3^, Taehoon Lee^4^, Gyusoo Bak^5^, Hanjun Cho^1^, Jong-Chan Park^1, 5^, Inki Kim^1, 2, 5, *^

^1^ Department of Biophysics, Institute of Quantum Biophysics, Sungkyunkwan University, Suwon 16419, Republic of Korea

^2^ Department of Intelligent Precision Healthcare Convergence, Sungkyunkwan University, Suwon 16419, Republic of Korea

^3^ Center for Quantum Technology, Korea Institute of Science and Technology (KIST), Seoul, 02792, Republic of Korea

^4^ Department of Biopharmaceutical Convergence, Sungkyunkwan University, Suwon 16419, Republic of Korea

^5^ Department of MetaBioHealth, Institute for Cross-disciplinary Studies (ICS), Sungkyunkwan University, Suwon 16419, Republic of Korea

*Corresponding author: [inki.kim@skku.edu](mailto:inki.kim@skku.edu)

# These authors contributed equally to the work

| **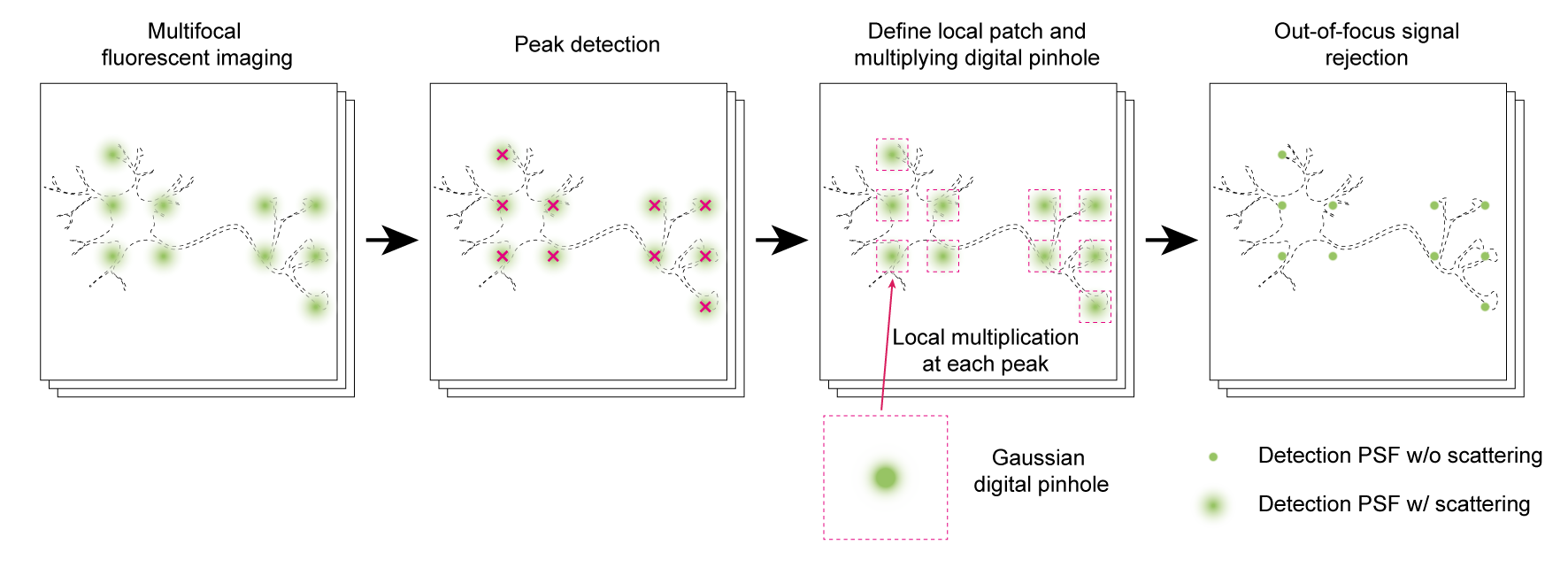** |
| --- |
| **Fig. S1 \| Schematic representation of digital pinholing.** Digital pinholing is an image processing technique that digitally applies an aperture to an image to reject out-of-focus signals. For ISM reconstruction, the precise peak position of the fluorescent signal under multifocal illumination must be identified using a peak detection algorithm. Then, local patches centered at the detected peaks are defined and multiplied by digital pinholes. The digital pinhole, defined within a specific patch size, functions as a localized filter to suppress out-of-focus signals around the peaks. We used Gaussian digital pinhole, which is widely used in digital pinholing; however, its profile may vary depending on the ISM reconstruction algorithm. Finally, an image with out-of-focus signals removed is obtained through local multiplication of digital pinhole patches. |
| 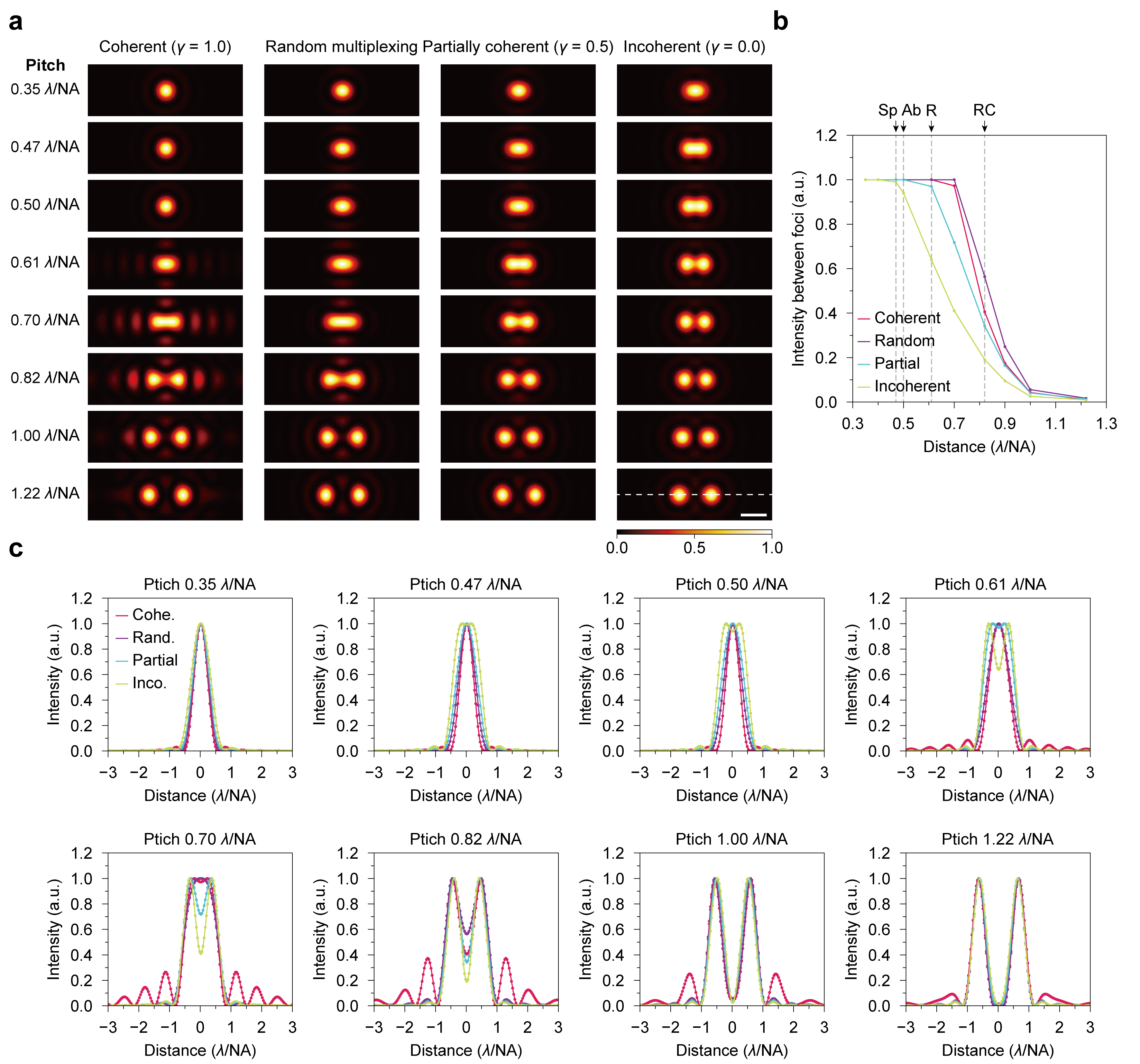 |
| **Fig. S2 \| Effect of index of coherence on interference.** (**a**) Simulation of interference between two foci at varying pitches (0.35–1.22 *λ*/NA) and indices of coherence (*γ*)^1^. The random multiplexing indicates that the foci were simulated with bifocal metalens designed using random multiplexing method. (**b**) Intensity values between foci with respect to the pitch. Sp, Sparrow^2^; Ab, Abbe; R, Rayleigh; RC, Rayleigh criterion for coherent light. (**c**) Horizontal intensity profiles from (a). Cohe., coherent; Rand., random multiplexing; Partial, partially coherent; Inco., incoherent. Scale bar: 1.00 *λ*/NA (a). |

| 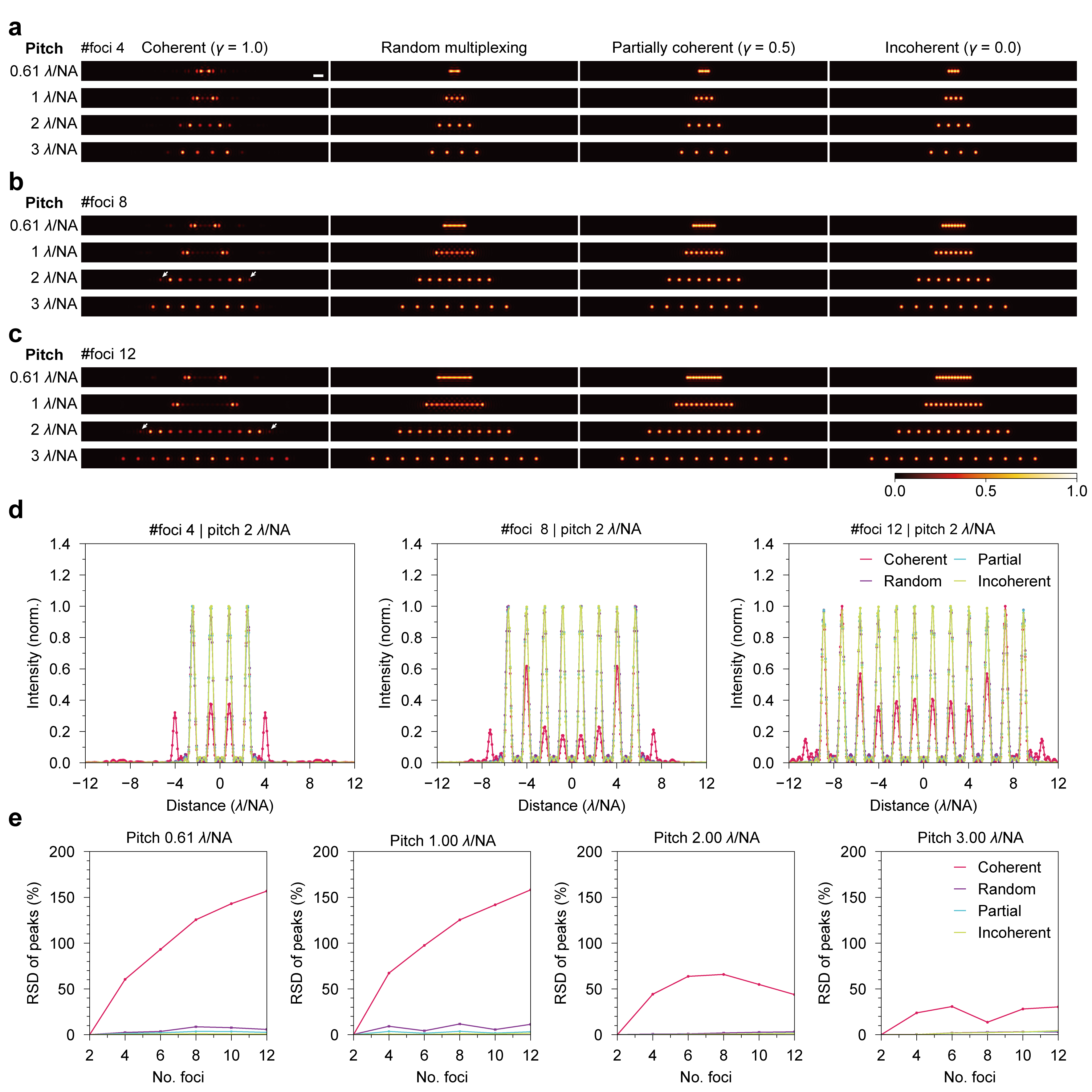 |
| --- |
| **Fig. S3 \| Comparative analysis of uniformity in 1D multifocal arrays.** (**a–c**) Simulation results of 1D multifocal arrays with varying numbers of foci (4, 8, and 12), pitches (0.61–3 *λ*/NA), and indices of coherence. The random multiplexing indicates that the foci were simulated with multifocal metalens designed using random multiplexing method. The white arrows indicate artifacts caused by interference. (**d**) Horizontal intensity profiles from (a–c). Coherently generated foci exhibited inhomogeneous peak intensity profiles due to the interference. (**e**) Uniformity in RSD with respect to the number of foci across various pitches (0.61–3 *λ*/NA) and indices of coherence. Notably, coherently generated foci exhibit larger RSD compared to others. Scale bar: 2 *λ*/NA (a–c). |

| **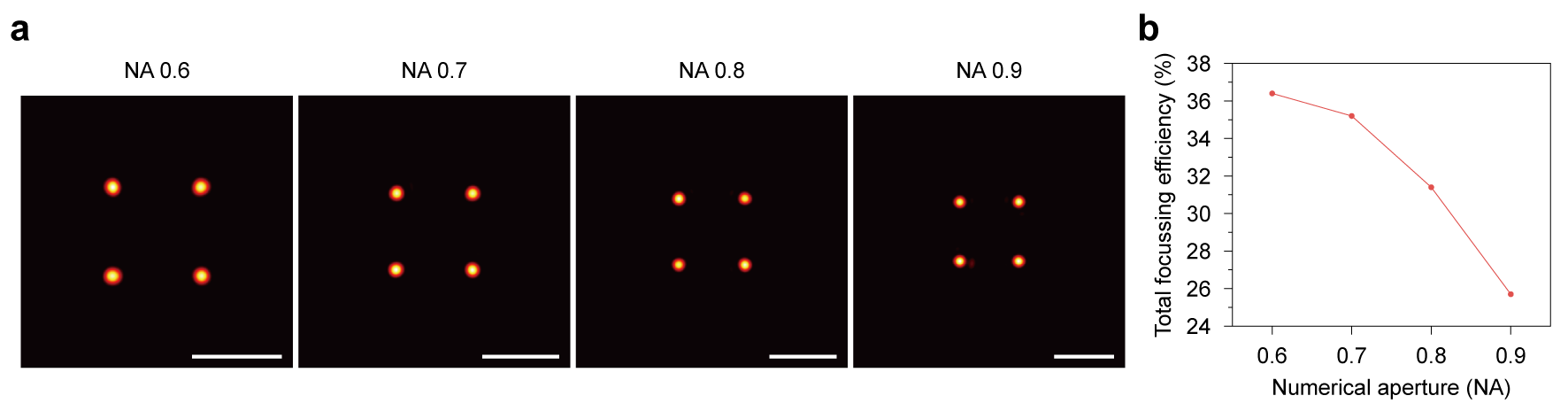** |
| --- |
| **Fig. S4 \| Focusing efficiency of multifocal metalens.** (**a**) FDTD simulation results of a 15 μm diameter multifocal metalens generating 4 foci at the focal plane. The metalenses were designed with a 3 AU pitch using hybrid multiplexing at various NAs. (**b**) Focusing efficiency of multifocal metalens with respect to NA. Scale bars: 3 AU (a). |

| **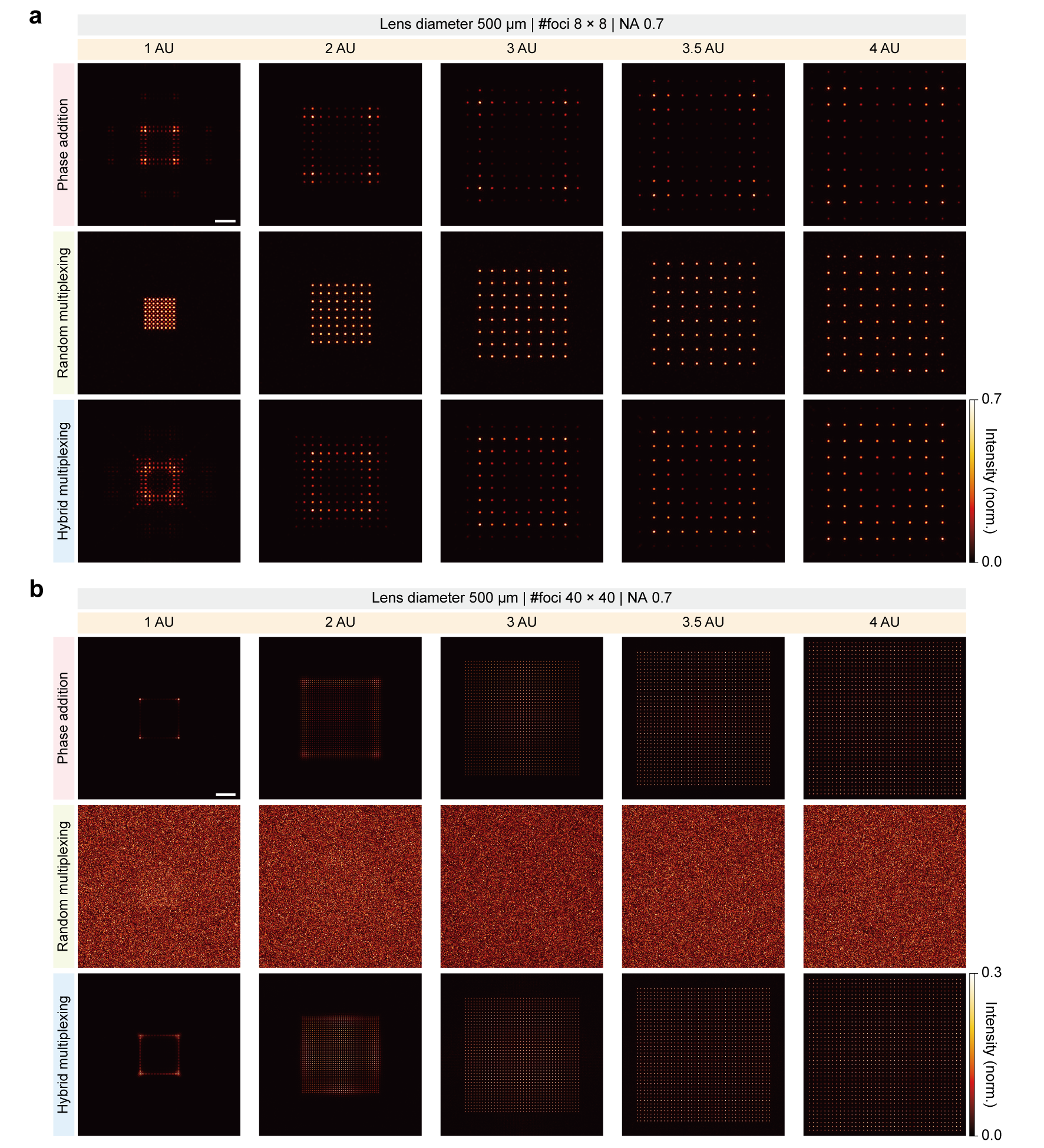** |
| --- |
| **Fig. S5 \| Simulation of multifocal arrays with varying pitches at fixed diameter, NA, and the number of foci.** (**a, b**) Simulated multifocal arrays using three different multiplexing methods with varying pitches (1–4 AU) and fixed foci of 8 × 8 (**a**) and 40 × 40 (**b**). The lens diameter and NA were set to 500 μm and 0.7, respectively. Scale bars: 5 AU (a) and 20 AU (b). |

| **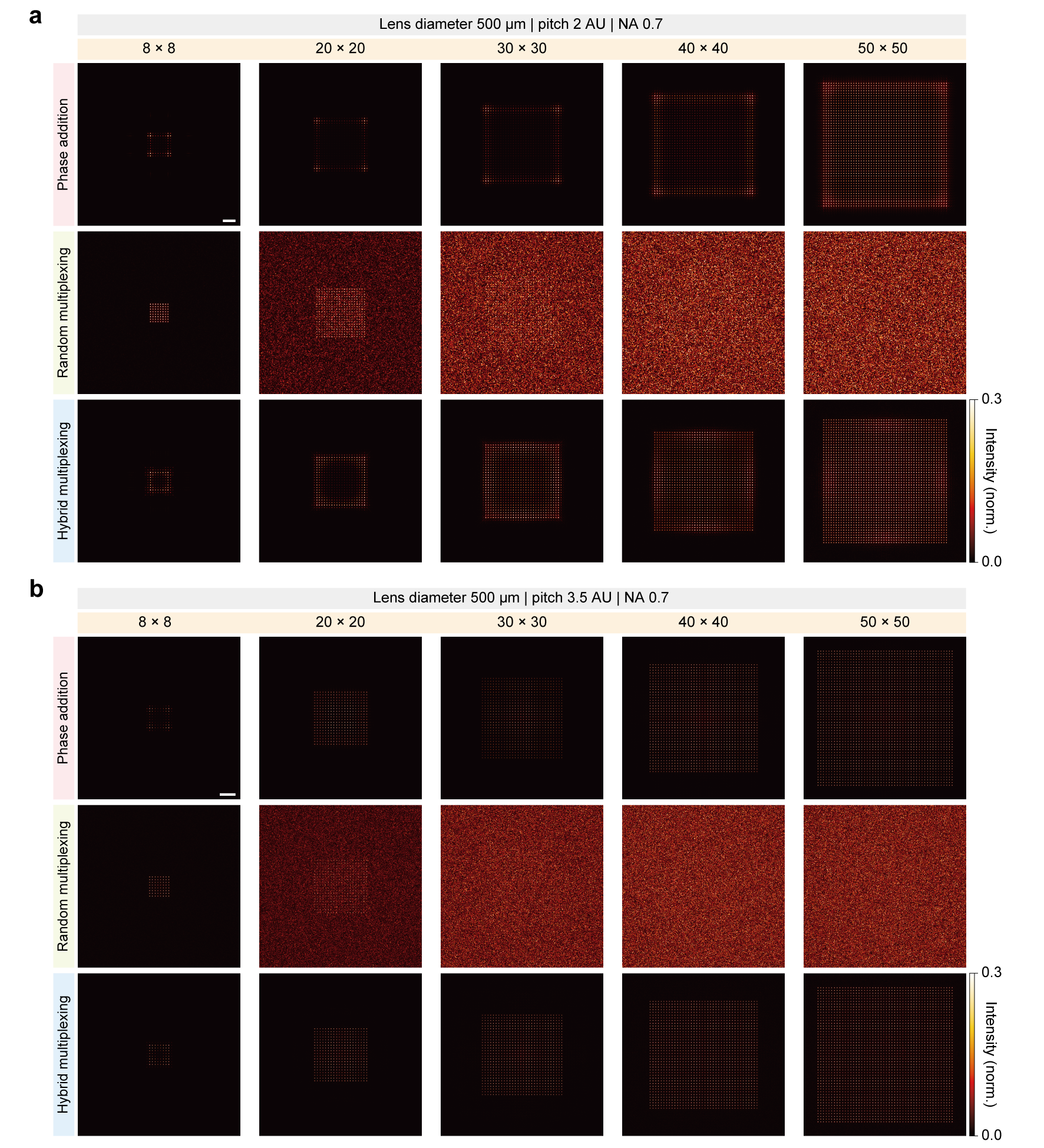** |
| --- |
| **Fig. S6 \| Simulation of multifocal arrays with varying numbers of foci at fixed diameter, NA, and pitch.** (**a, b**) Simulated multifocal arrays using three different multiplexing methods with varying numbers of foci (from 8 × 8 to 50 × 50) and fixed pitches of 2 AU (**a**) and 3.5 AU (**b**). The lens diameter and NA were set to 500 μm and 0.7, respectively. Scale bars: 10 AU (a) and 20 AU (b). |

| 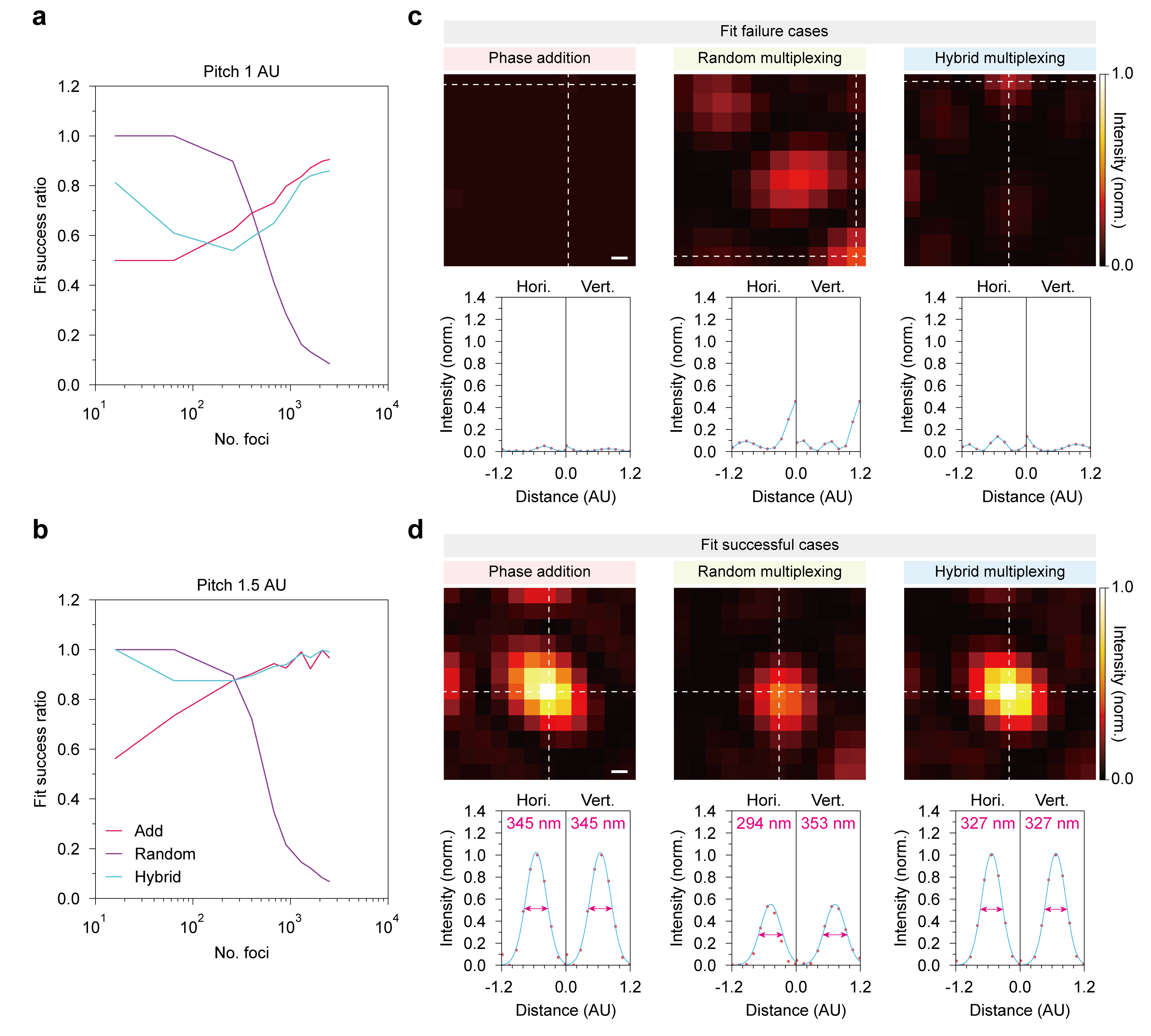 |
| --- |
| **Fig. S7 \| Fit success ratio across different multiplexing methods.** (**a, b**) Fit success ratio with respect to the number of foci for three different multiplexing methods at pitches of 1 AU (**a**) and 1.5 AU (**b**), with fixed diameter 500 μm. (**c, d**) Representative images showing horizontal (Hori.) and vertical (Vert.) intensity profiles of failure fit (**c**) and successful fit (**d**) to Gaussian function. Scale bars: 100 nm (c, d). |

| 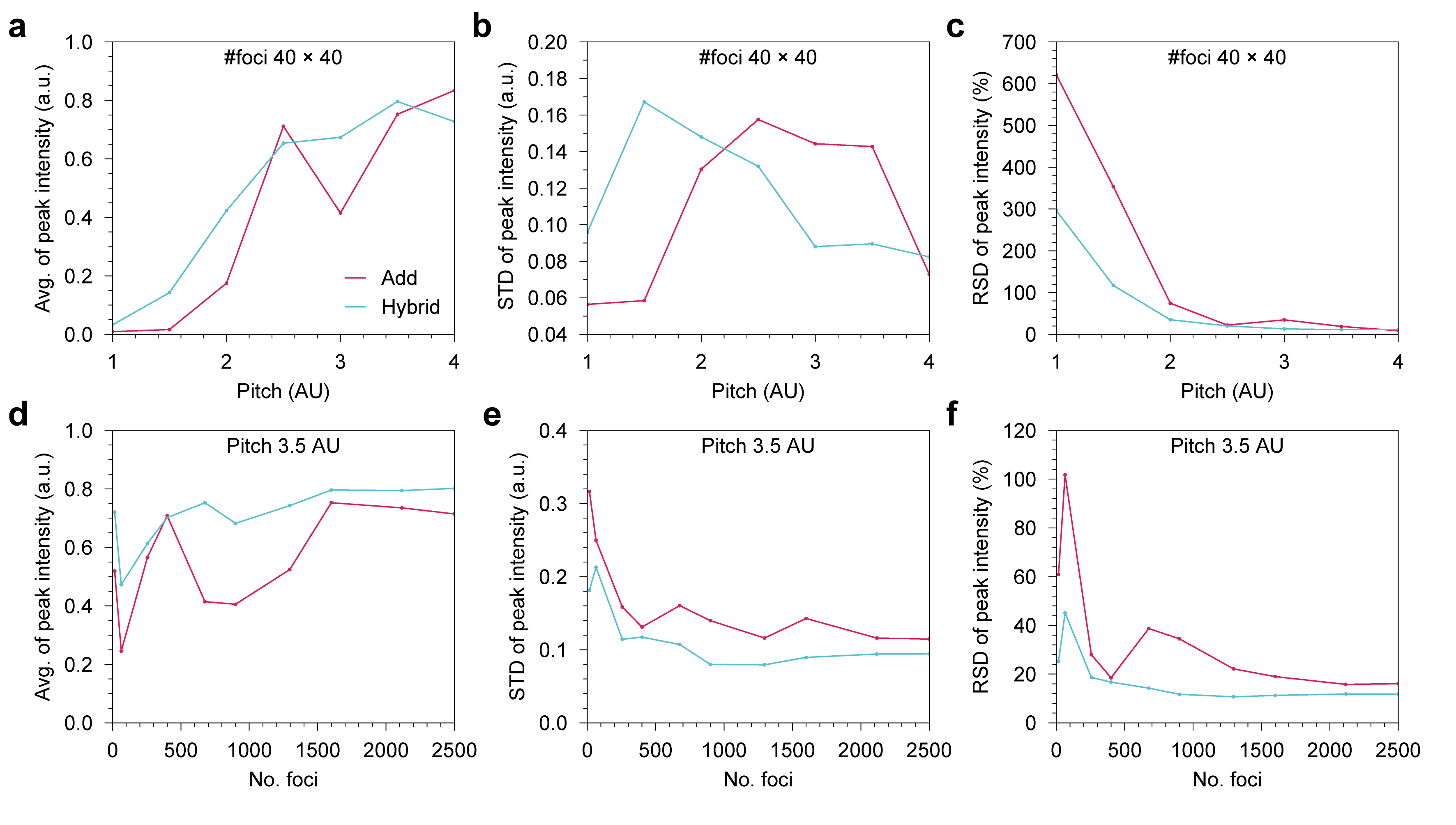 |
| --- |
| **Fig. S8 \|** **Quantitative analysis of simulated multifocal array quality with respect to pitch and the number of foci.** (**a–c**) Average peak intensity (**a**), STD (**b**), and RSD (**c**) of the multifocal array with respect to the pitch, with a fixed diameter of 500 μm, NA of 0.7, and 40 × 40 foci. (**d–f**) Average peak intensity (**d**), STD (**e**), and RSD (**f**) of the multifocal array with respect to the number of foci, with a fixed diameter of 500 μm, NA of 0.7, and a pitch of 3.5 AU. |

| **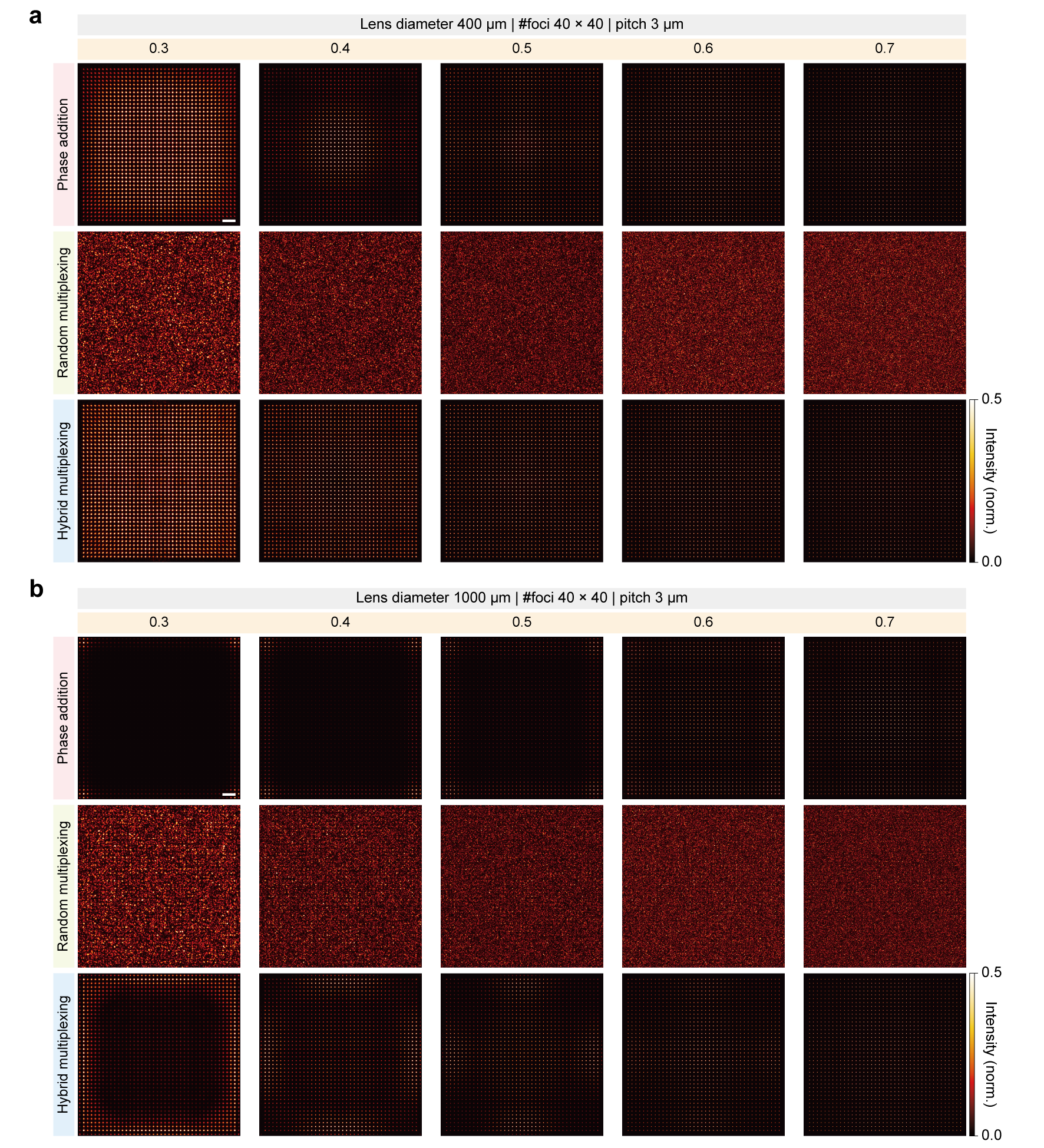** |
| --- |
| **Fig. S9 \| Simulation of multifocal arrays with varying NAs at fixed diameter, pitch, and the number of foci.** (**a, b**) Simulated multifocal arrays using three different multiplexing methods with varying NAs (0.3–0.7) and fixed diameters of 400 μm (**a**) and 1000 μm (**b**). The number of foci and pitch were set to 40 × 40 and 3 μm, respectively. Scale bars: 10 μm (a, b). |

| **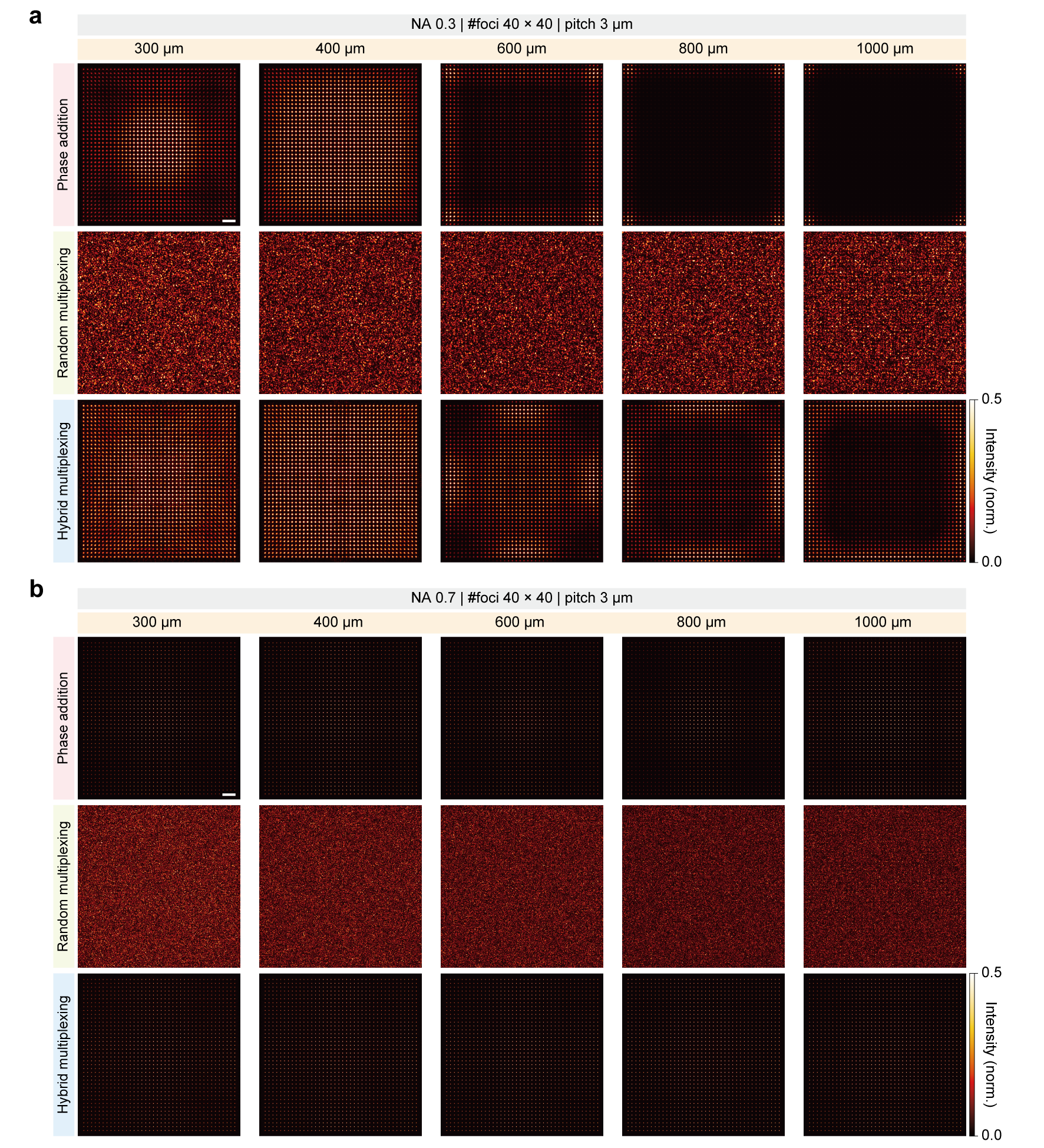** |
| --- |
| **Fig. S10 \| Simulation of multifocal arrays with varying diameters at fixed NA, pitch, and the number of foci.** (**a, b**) Simulated multifocal arrays using three different multiplexing methods with varying diameters (300–1000 μm) and fixed NA of 0.3 (**a**) and 0.7 (**b**). The number of foci and pitch were set to 40 × 40 and 3 μm, respectively. Scale bars: 10 μm (a, b). |

| 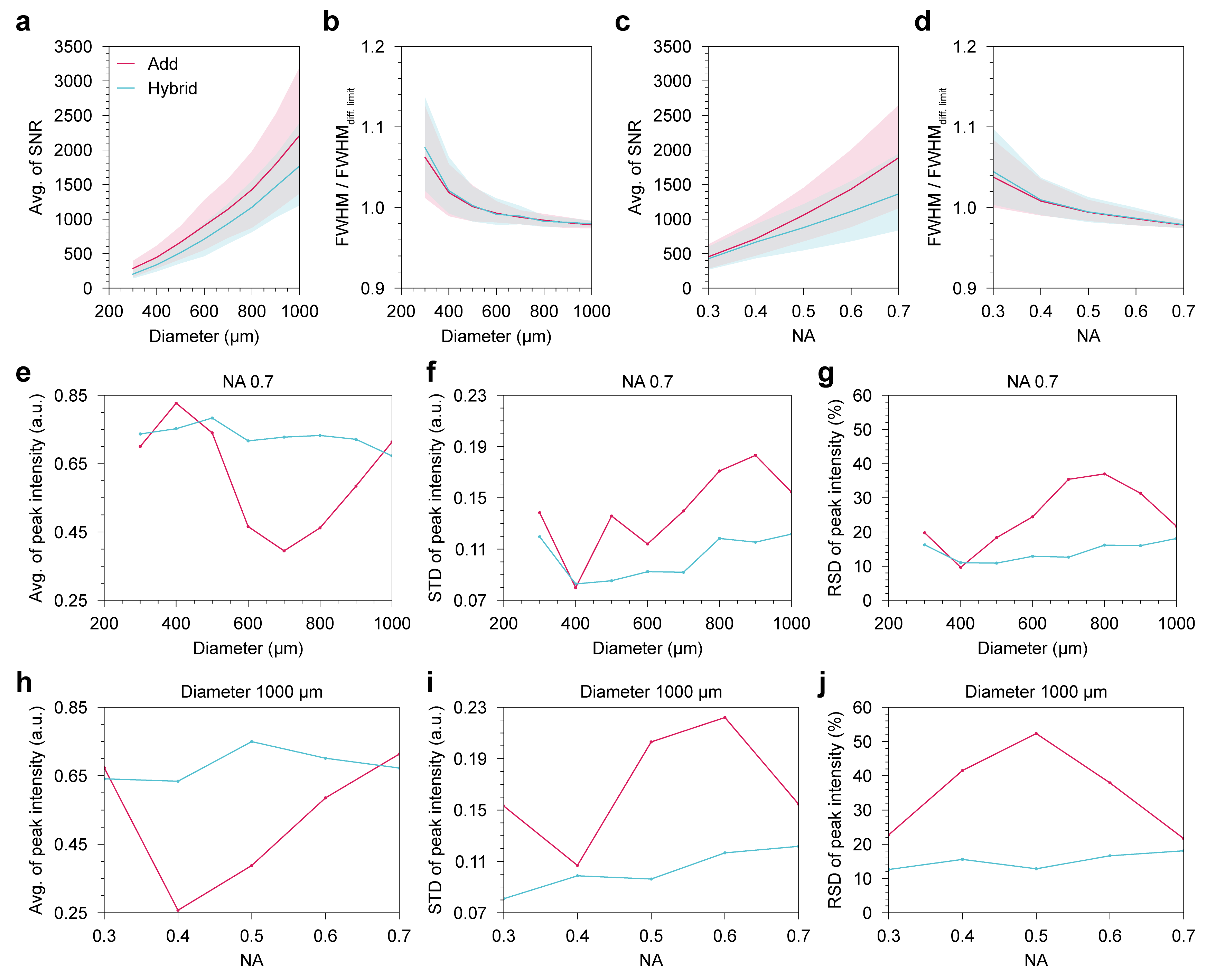 |
| --- |
| **Fig. S11 \|** **Quantitative analysis of simulated multifocal array quality with respect to diameter and NA.** (**a, b**) SNR (**a**) and FWHM normalized by the theoretical FWHM (**b**) of multifocal arrays with respect to the diameter, with a fixed pitch of 3.5 AU and 40 × 40 foci. (**c, d**) SNR (**c**) and FWHM normalized by the theoretical FWHM (**d**) of multifocal arrays with respect to the NA, with a fixed pitch of 3.5 AU and 40 × 40 foci. (**e–g**) Average peak intensity (**e**), STD (**f**), and RSD (**g**) of the multifocal array with respect to diameter, with a fixed NA of 0.7, 40 × 40 foci, and a pitch of 3.5 AU. (**h–j**) Average peak intensity (**h**), STD (**i**), and RSD (**j**) of the multifocal array with respect to NA, with a fixed diameter of 1000 μm, 40 × 40 foci, and a pitch of 3.5 AU. Error bars in (a–d) represent the STD. |

| 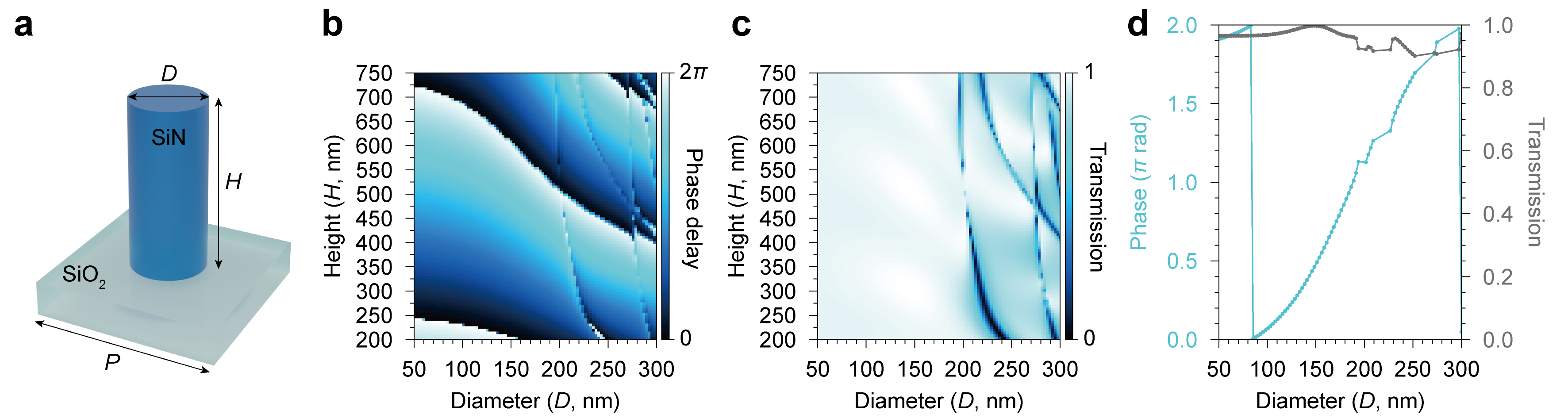 |
| --- |
| **Fig. S12 \| RCWA simulation results for creating SiN meta-atom library.** (**a**) Geometrical parameters of cylindrical SiN meta-atom on the SiO_2_ substrate. *D*, diameter; *H*, height; *P*, period. A period of 330 nm was used for this simulation. (**b, c**) Phase delay (**b**) and transmission (**c**) of the SiN meta-atoms with varying diameters (50–305 nm) and heights (200–750 nm). (**d**) SiN meta-atoms with a height of 750 nm were adopted, covering whole 2*π* phase range with high transmission (>90%). |

| 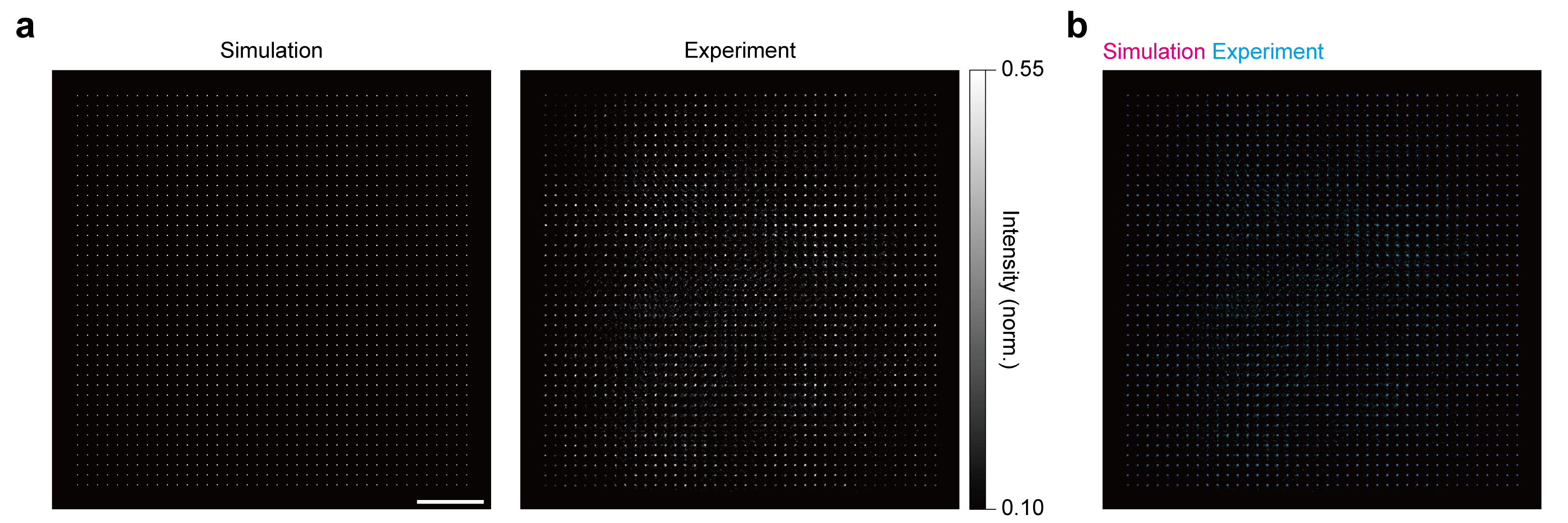 |
| --- |
| **Fig. S13 \| Comparison of simulated and experimentally obtained PSFs.** (**a**) Simulated (left) and experimentally measured (right) multifocal arrays. The metalens was designed to have parameters of 40 × 40 foci, a pitch of 3.5 AU, NA of 0.7, and a diameter of 1000 μm. (**b**) Overlay of the simulated (magenta) and experimental (cyan) results from (a). Scale bar: 20 μm (a, b). |

| 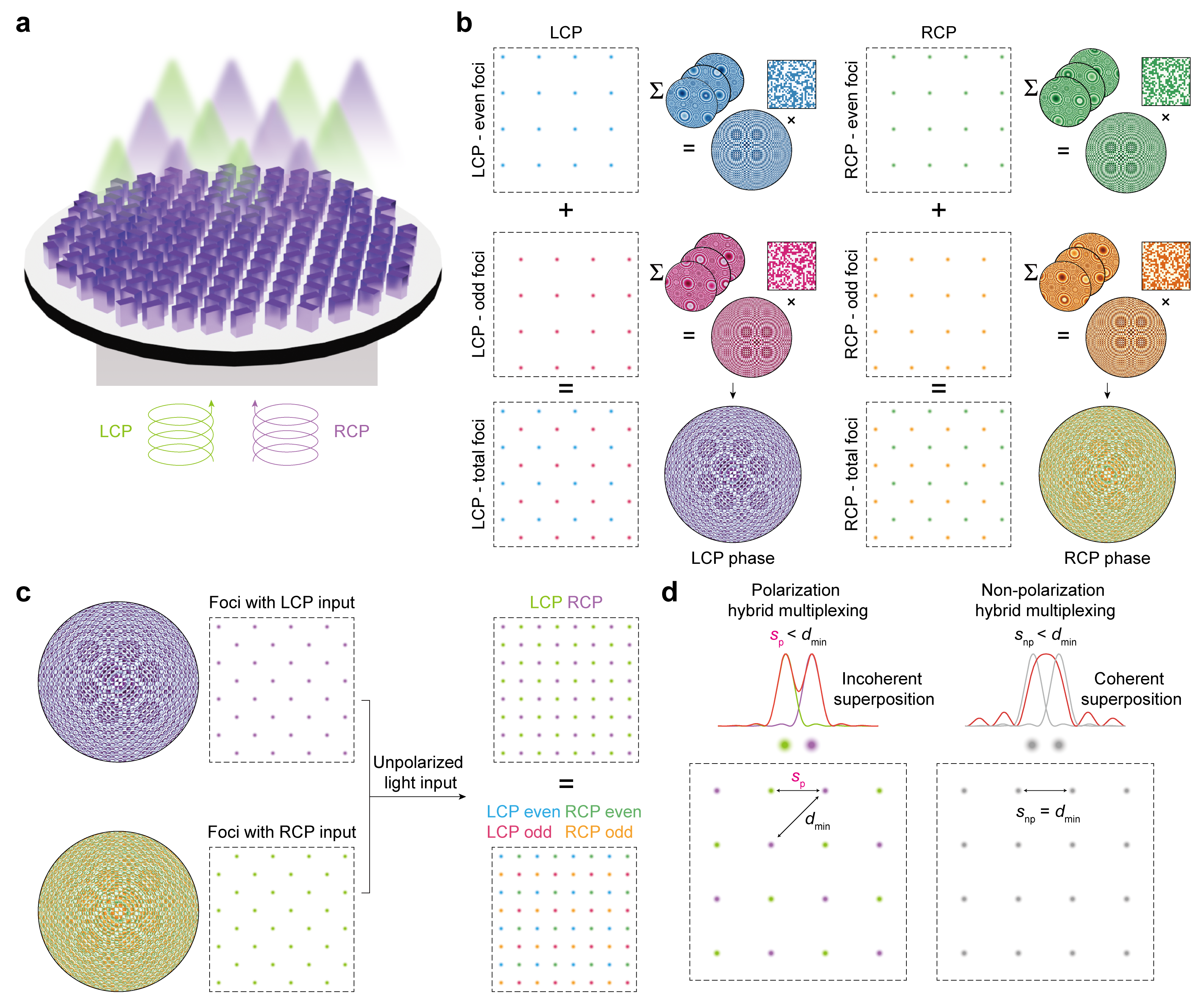 |
| --- |
| **Fig. S14 \| Principle of polarization (pol-) hybrid multiplexing.** (**a**) Schematic of the polarization-dependent multifocal metalens using pol-hybrid multiplexing. (**b**) Design workflow of multifocal metalens employing the pol-hybrid multiplexing method. This process combines the hybrid multiplexing technique with independent phase control of orthogonally polarized light using polarization-dependent meta-atoms. (**c**) Expected outcomes of multifocal arrays under LCP, RCP, and unpolarized light input. (**d**) Comparison between pol-hybrid multiplexing and non-polarization hybrid multiplexing. Here, $d_{\min}$ and $s$ represent the minimum pitch that does not significantly interfere between adjacent foci and pitch of the multifocal array, respectively. The subscripts ‘p’ and ‘np’ indicate polarization and non-polarization hybrid multiplexing, respectively. In the case of non-polarization, $s_{\mathrm{np}}$ can only be reduced a distance comparable to $d_{m\mathrm{in}}$ because adjacent foci coherently interfere and superimpose at distances smaller than $d_{m\mathrm{in}}$. However, smaller $s_{p}$ than $d_{\min}$ is achievable with pol-hybridization multiplexing, as adjacent foci incoherently superimpose, making it less sensitive to interference. |

| 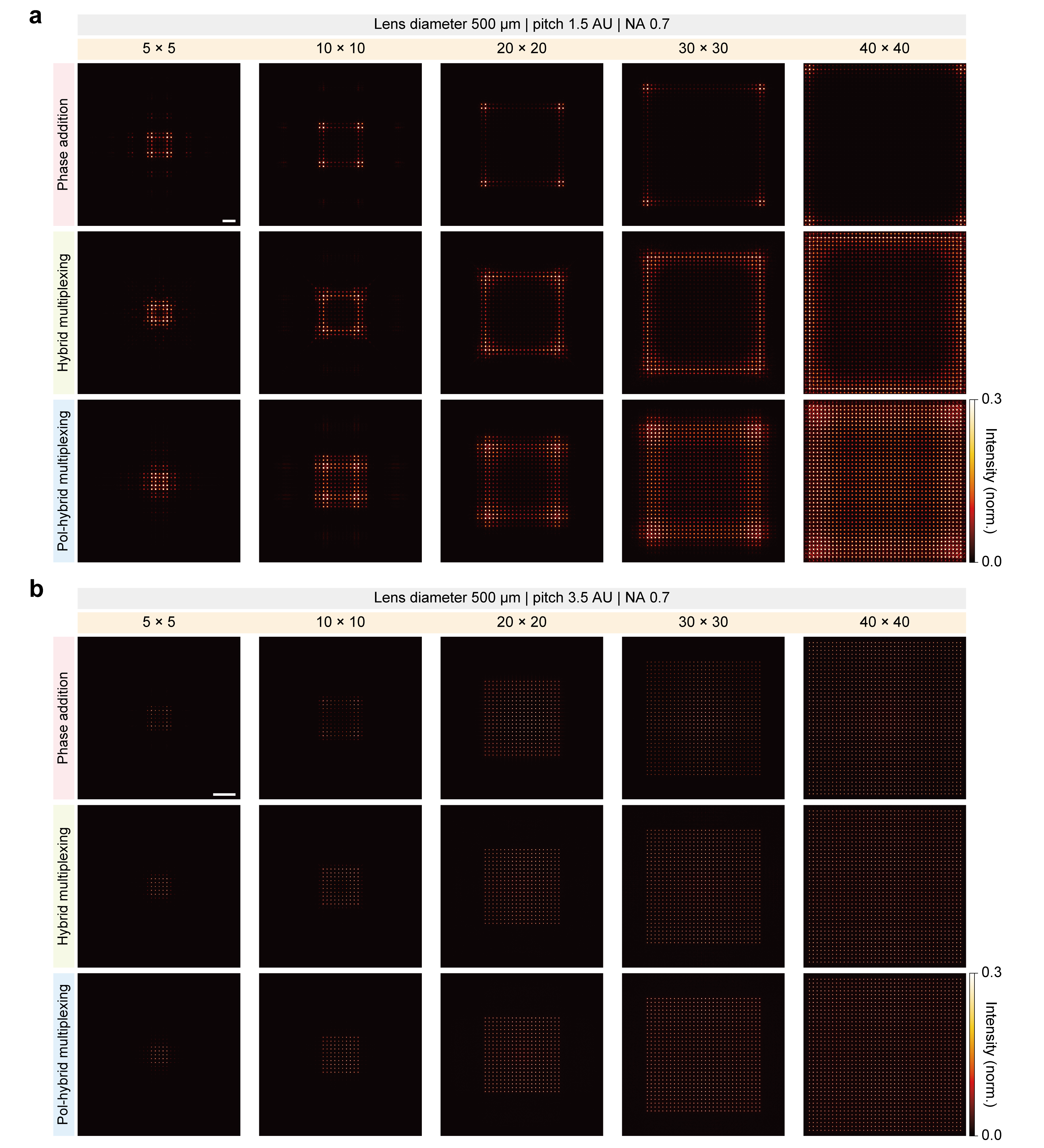 |
| --- |
| **Fig. S15 \| Simulation of pol-hybrid multiplexing with varying numbers of foci at fixed NA, pitch, and diameter.** (**a, b**) Simulated multifocal arrays using three different multiplexing methods, including pol-hybrid multiplexing, with varying numbers of foci (from 5 × 5 to 40 × 40) and fixed pitches of 1.5 AU (**a**) and 3.5 AU (**b**). The diameter and NA were set to 500 μm and 0.7, respectively. Scale bars: 5 AU (a) and 20 AU (b). |

| 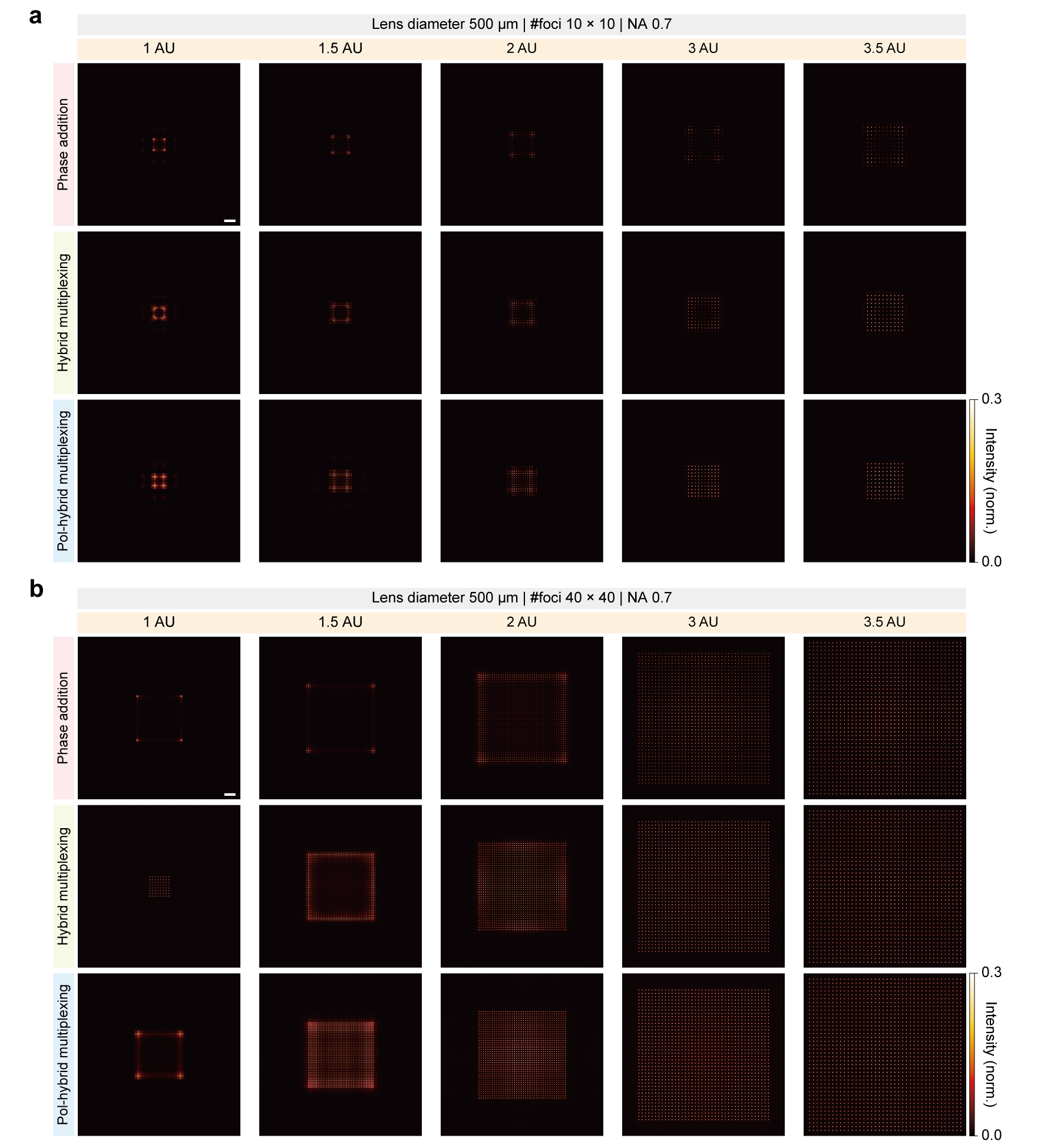 |
| --- |
| **Fig. S16 \| Simulation of pol-hybrid multiplexing with varying pitches at fixed NA, diameter, and the number of foci.** (**a, b**) Simulated multifocal arrays using three different multiplexing methods, including pol-hybrid multiplexing, with varying pitches (1–3.5 AU) and fixed foci of 10 × 10 (**a**) and 40 × 40 (**b**). The diameter and NA were set to 500 μm and 0.7, respectively. Scale bars: 10 AU (a, b). |

| **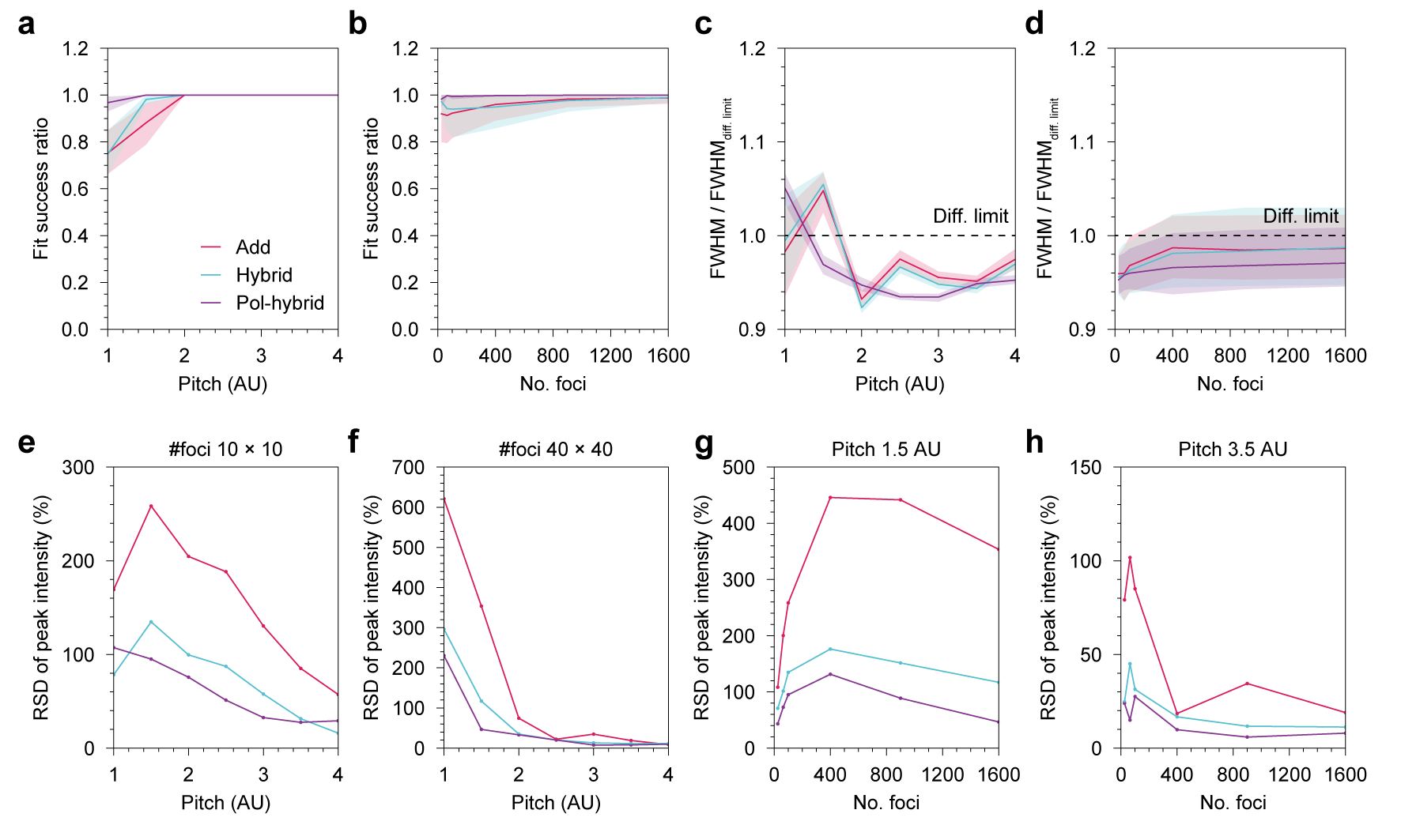** |
| --- |
| **Fig. S17 \|** **Quantitative analysis of the quality of simulated pol-hybridization multifocal arrays.** (**a, b**) Fit success ratio with respect to pitch (**a**) and number of foci (**b**). (**c, d**) FWHM normalized by the theoretical value with respect to pitch (**c**) and number of foci (**d**). (**e, f**) RSD with respect to pitch at fixed foci of 10 × 10 (**e**) and 40 × 40 (**f**). (**g, h**) RSD with respect to the number of foci at fixed pitches of 1.5 AU (**g**) and 3.5 AU (**h**). The diameter and NA were set to 500 µm and 0.7, respectively, throughout these simulations. Error bars in (a–d) represent the STD. |

| 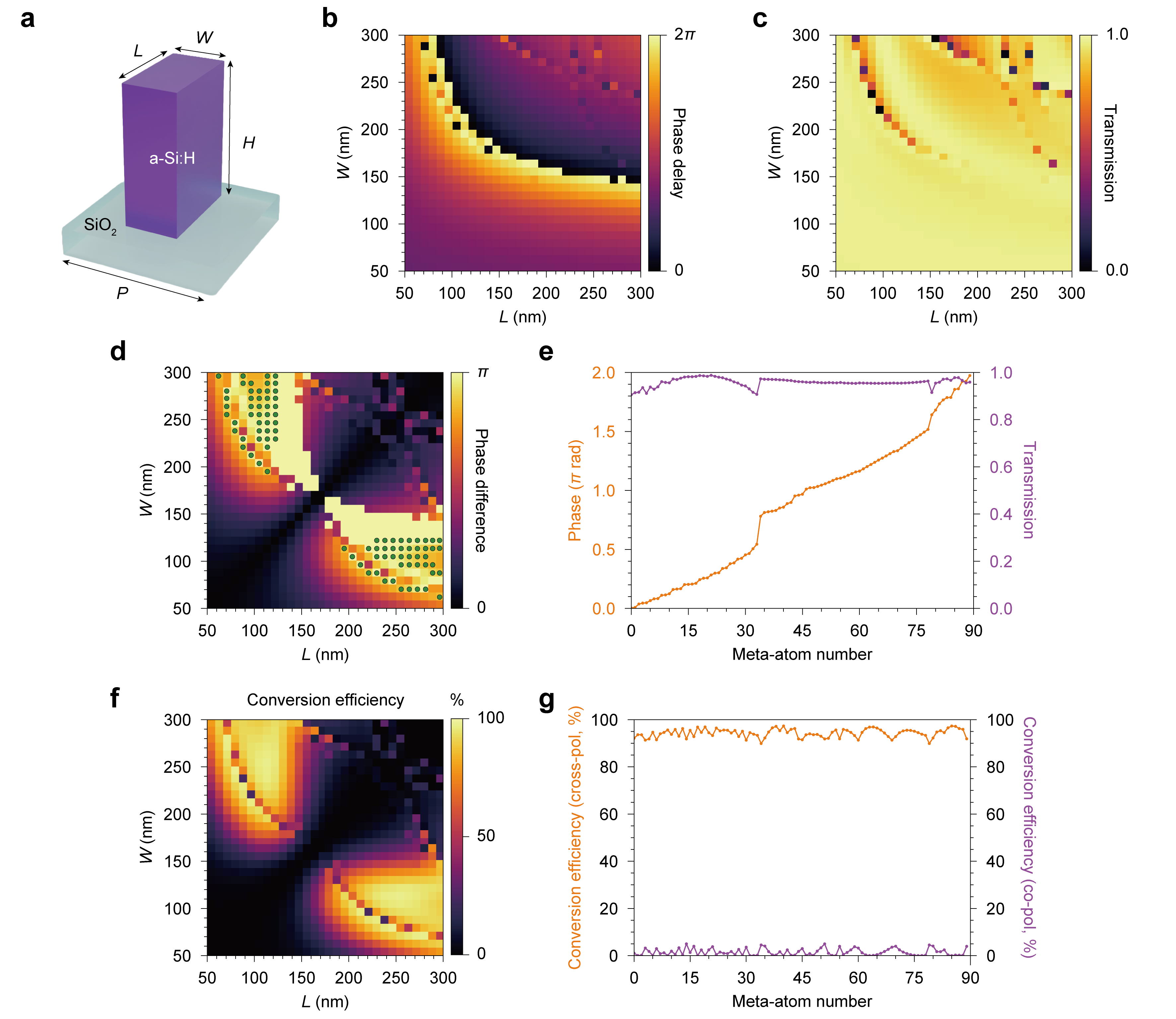 |
| --- |
| **Fig. S18 \| RCWA simulation results for creating a-Si:H meta-atom library.** (**a**) Geometrical parameters of rectangular a-Si:H meta-atom on the SiO_2_ substrate. *W*, width; *L*, length; *H*, height; *P*, period. The period of 350 nm and height of 650 nm were used for this simulation. (**b, c**) Phase delay (**b**) and transmission (**c**) of the a-Si:H meta-atoms with varying widths (50–300 nm) and lengths (50–300 nm). (**d**) Difference in phase delay under the incidence of *x*- and *y*-polarized light. Green dots represent selected meta-atoms that satisfy a phase difference of *π* rad, with an error of ±15% and a transmittance greater than 90%. (**e**) Phase delay and transmission profiles of the adopted meta-atoms, covering the entire 2*π* phase range with high transmission (>90%). (**f**) Cross-polarization conversion efficiency of each simulated meta-atoms. (**g**) Cross- and co-polarization conversion efficiencies of the selected meta-atoms. |

| 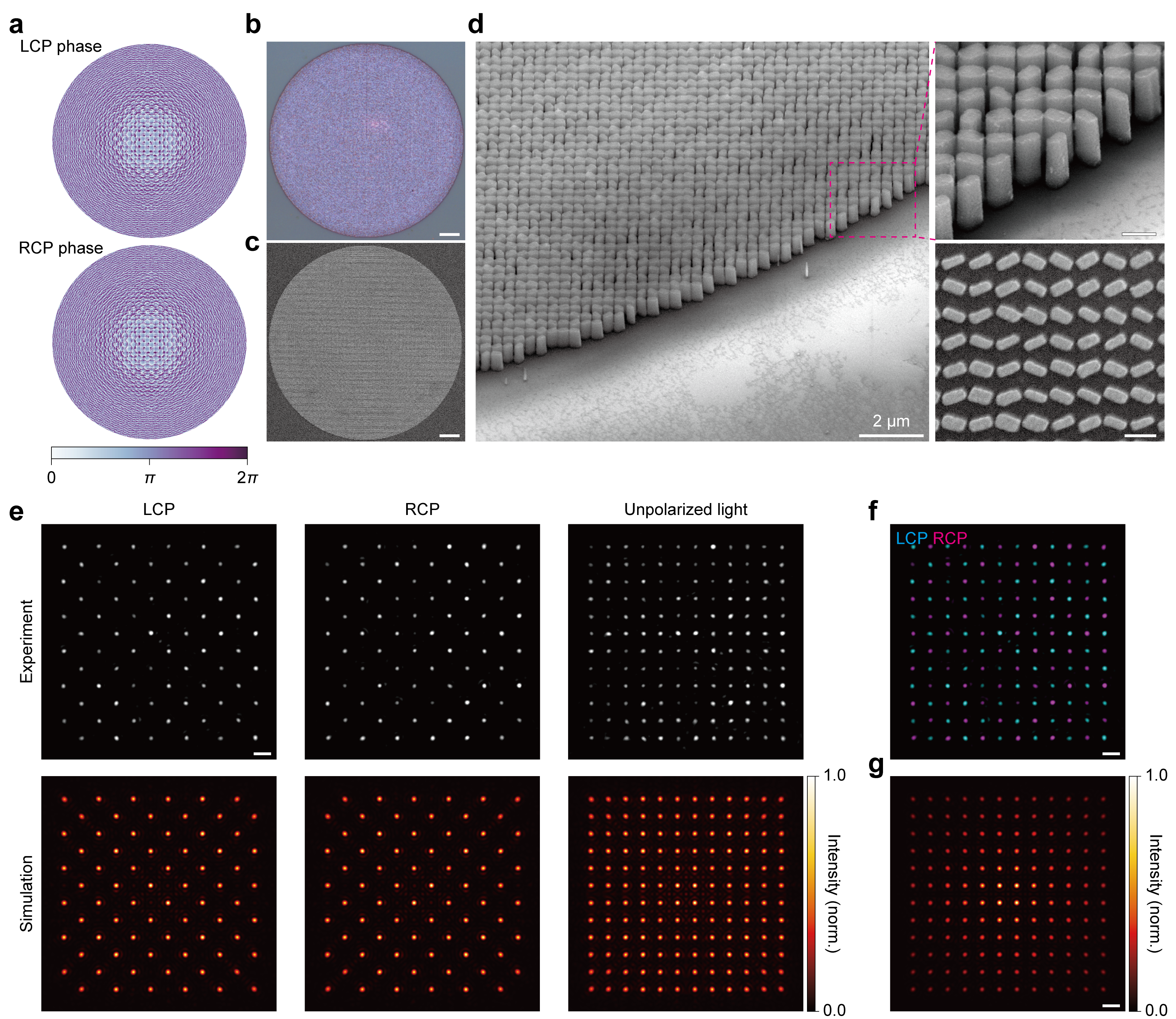 |
| --- |
| **Fig. S19 \| Fabrication of a multifocal metalens using pol-hybrid multiplexing, operating at 633 nm.** (**a**) The LCP and RCP phase profiles of the multifocal metalens, designed to create 12 × 12 foci with a pitch of 2 AU and NA of 0.7 under unpolarized light illumination at 633 nm. The lens diameter is 100 μm. (**b**) Optical microscopy image of the fabricated multifocal metalens. (**c**) SEM image of the entire view of the fabricated multifocal metalens. (**d**) SEM image of the edge of the metalens (left) and a zoomed-in view (top right). The image at the bottom right shows a top view of the meta-atoms in the fabricated metalens. (**e**) Multifocal arrays obtained experimentally and through simulation under the incidence of LCP, RCP, and unpolarized light. (**f**) An overlay of the multifocal array images generated under illumination of LCP (cyan) and RCP (magenta) light shown in (e). (**g**) Simulated multifocal arrays using hybrid multiplexing methods without polarization modulation, with same design parameters of 12 × 12 foci, a pitch of 2 AU, and NA of 0.7. Scale bars: 10 μm (b, c), 400 nm (d, top and bottom right), and 2 AU (e–g). |

| **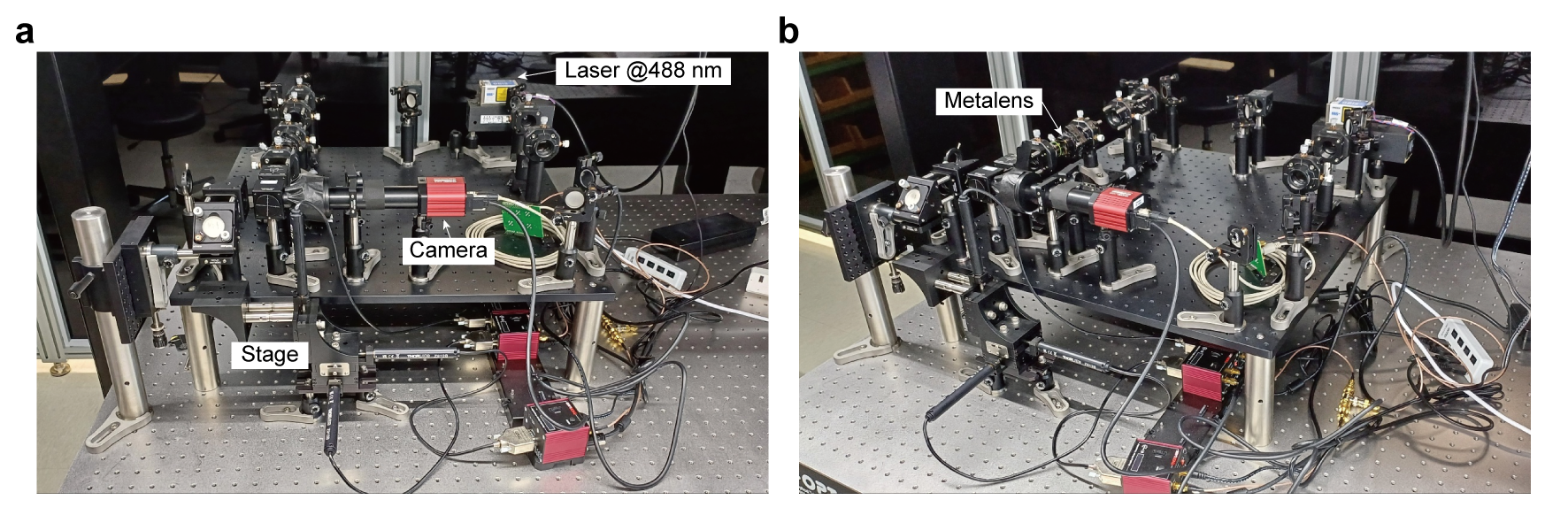** |
| --- |
| **Fig. S20 \| Experimental setup of MMISM.** (**a, b**) Front view (**a**) and side view (**b**) of the MMISM setup. |

| **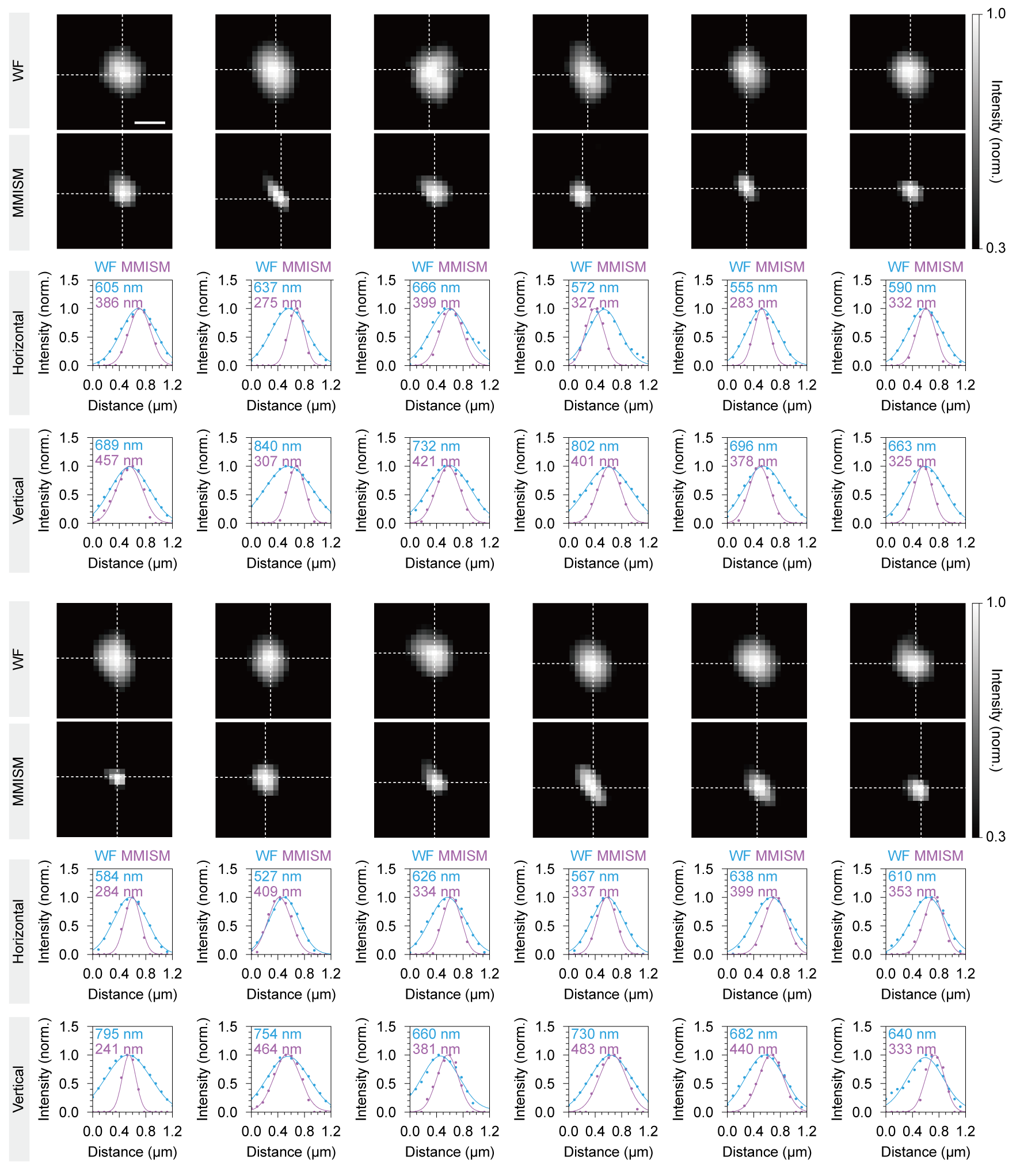** |
| --- |
| **Fig. S21 \| Measurements of resolution I.** Images of 30 nm fluorescent beads with their corresponding horizontal and vertical intensity profiles. The numbers above the intensity profiles indicate the FWHM of each profile for WF (light blue) and MMISM (purple). Scale bar: 500 nm. |

| **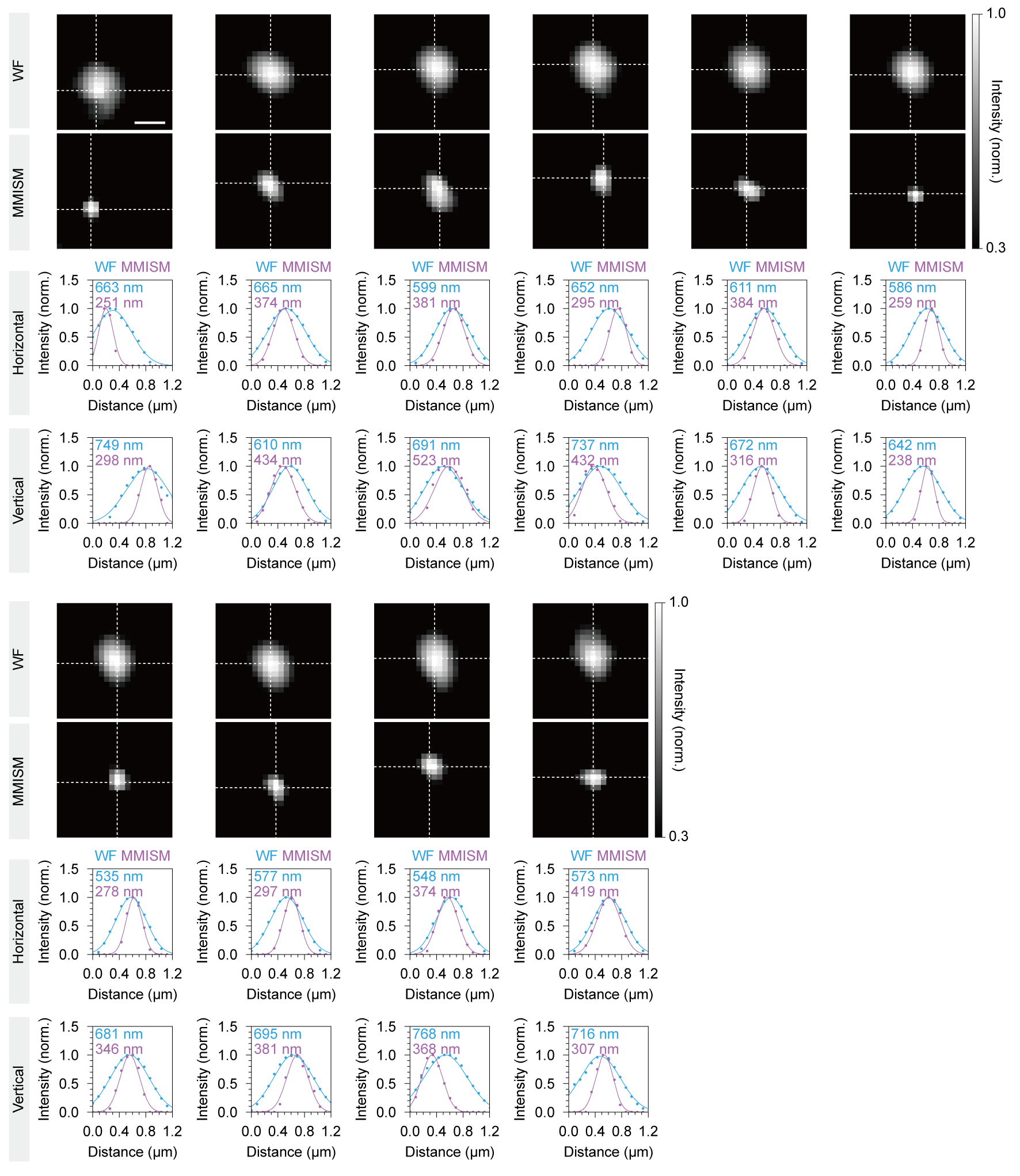** |
| --- |
| **Fig. S22 \| Measurements of resolution II.** Images of 30 nm fluorescent beads with their corresponding horizontal and vertical intensity profiles. The numbers above the intensity profiles indicate the FWHM of each profile for WF (light blue) and MMISM (purple). Scale bar: 500 nm. |

| **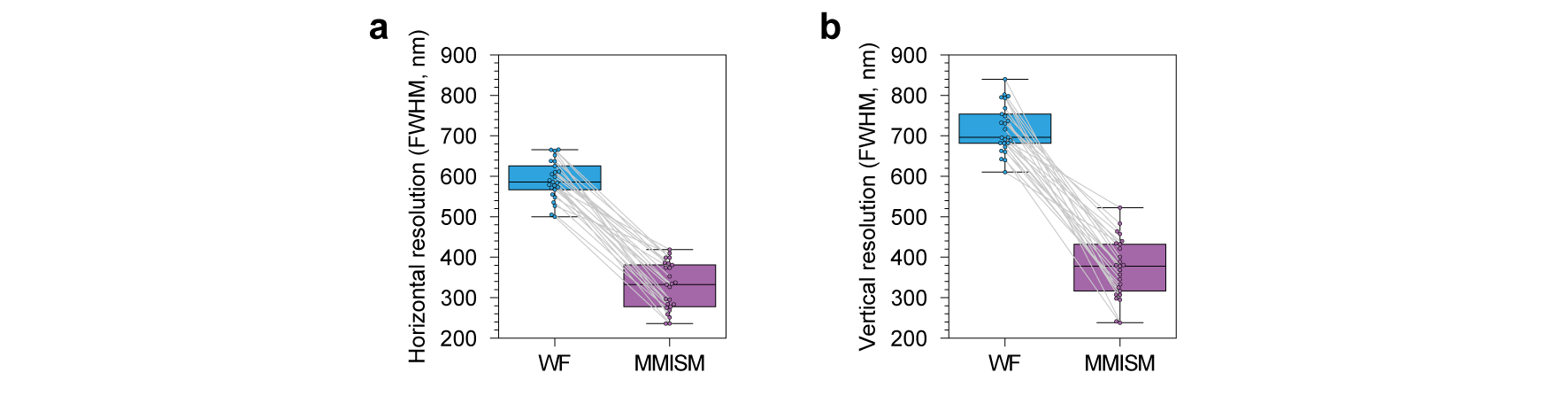** |
| --- |
| **Fig. S23 \| Quantification of resolution. (a, b)** Resolution quantification measured in horizontal (**a**) and vertical (**b**) directions. The paired individual points connected by gray lines for WF and MMISM represent measurements from single microbeads. A total of 25 microbeads were analyzed, including the 3 beads shown in Fig. 4b–d. The average horizontal resolutions for WF and MMISM were 591 nm and 327 nm, respectively, while the average vertical resolutions were 717 nm and 373 nm, respectively. |

| **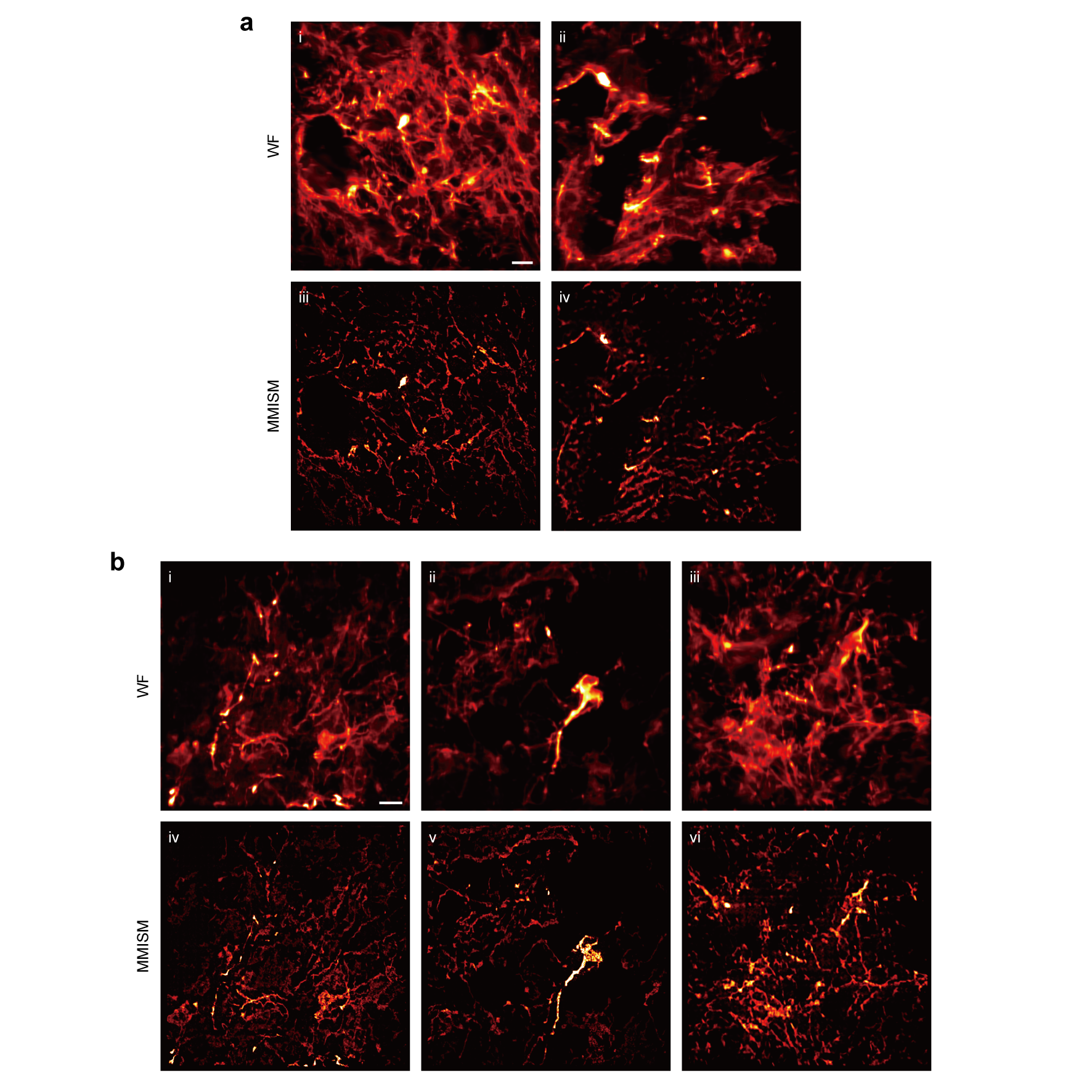** |
| --- |
| **Fig. S24 \| WF and MMISM images of forebrain organoid samples stained with pTau and MAP2 markers.** (**a**) WF (i, ii) and MMISM (iii, iv) images of the sectioned forebrain organoid samples stained with pTau marker. (**b**) WF (i–iii) and MMISM (iv–vi) images of the section forebrain organoid samples stained with MAP2 marker. Scale bars: 10 μm (a, b). |

Supplementary Notes 1. Superposition of fields with arbitrary coherence

According to superposition principle^1,3,4^, the superposition of optical fields can be represented as follows:

|  | $\psi=\sum_{i=1}^{F} \psi_{i}$ | (S1) |
| --- | --- | --- |

where $\psi$ denotes the optical field in phasor form, and $F$ is the number of fields. For $F=2$, the intensity of the superimposed field is described as $\left| \psi\right|^{2}=\left| \psi_{1} \right|^{2}+\left| \psi_{2} \right|^{2}+\left( {\psi_{1}^{*}\psi}_{2}+\psi_{1}\psi_{2}^{*} \right)$. This represents the intensity of individual fields ($\left| \psi_{1} \right|^{2}+\left| \psi_{2} \right|^{2})$, plus interference terms $\left( {\psi_{1}^{*}\psi}_{2}+\psi_{1}\psi_{2}^{*} \right)$. If the fields are incoherent, then the interference terms should be zero. To generalize the superposition of fields based on the degree of coherence, the index of coherence (*γ*) can be introduced^1^. Let $P_{\mathrm{coh}}=\left| \sum_{i=1}^{n} \psi_{i} \right|^{2}$ and $P_{\mathrm{inco}}=\sum_{i=1}^{n} \left| \psi_{i} \right|^{2}$ represent the intensities of coherently and incoherently superimposed fields, respectively (**equation (S1)**). The intensity of the superimposed fields with arbitrary coherence can be described using *γ* as follows:

|  | $P_{\mathrm{int}}=P_{\mathrm{coh}}-P_{\mathrm{inco}}$ | (S2) |
| --- | --- | --- |
|  | $P=P_{\mathrm{inco}}+\gamma P_{\mathrm{int}}$  ,$\mathrm{where} P=\left\{ \begin{aligned} P_{\mathrm{coh}} \mathrm{if} \gamma=1 \\ P_{\mathrm{part}} \mathrm{if} 0<\gamma<1 \\ P_{\mathrm{inco}} \mathrm{if} \gamma=0 \end{aligned} \right., 0\leq\gamma\leq1$ | (S3) |

where $P$ is the generalized notation for the intensity of the superimposed fields, $P_{\mathrm{int}}$ is the intensity contribution from interference, and $P_{\mathrm{part}}$ is the resulting intensity of the superimposed fields with partial coherence. In this study, *γ* values of 1, 0.5, and 0 were used to simulate coherent, partially coherent, and incoherent superpositions (**equation (S1), (S2)**).

Supplementary Notes 2. Phase addition multiplexing

| 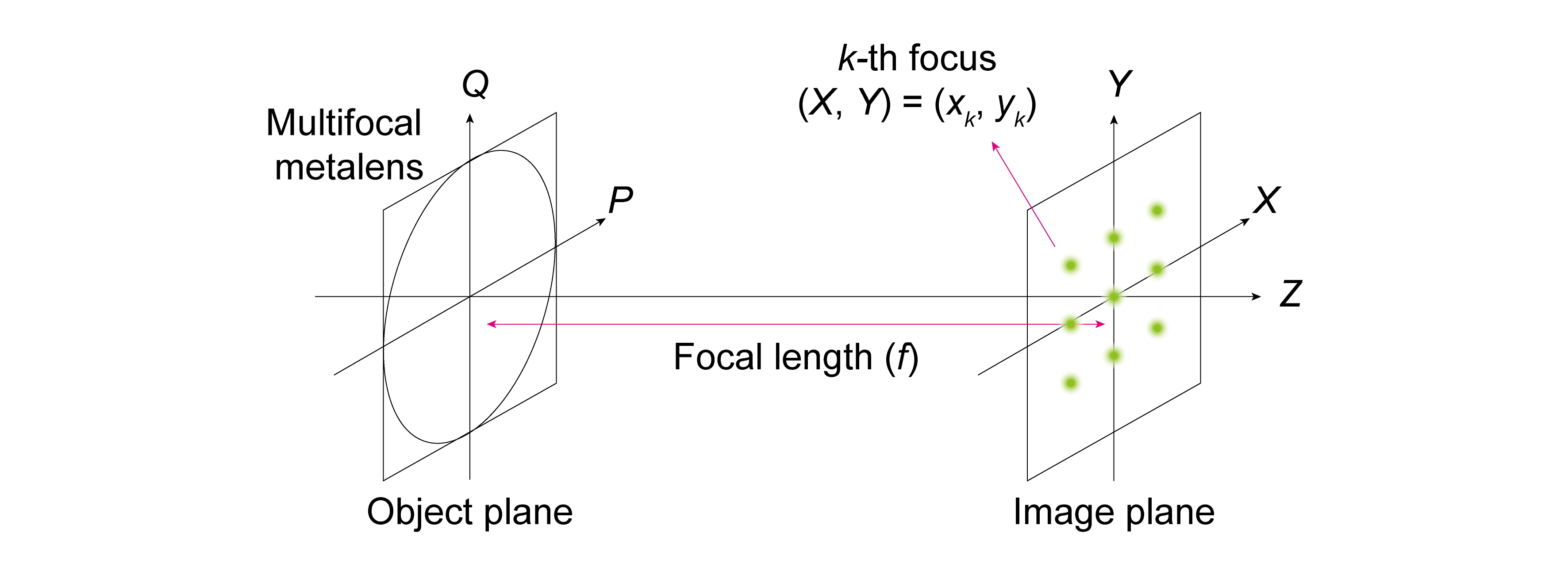 |
| --- |
| **Fig. S25 \| Coordinates system for multifocal metalens.** Upon light illumination on the multifocal metalens in the object plane, the propagated field forms a multifocal array in the image plane. The *k*-th focus is formed at the coordinate (*x_k_*, *y_k_*). |

Let $t_{k}\left( p, q \right)=e^{i\varphi_{k}}$ represent the transmittance function of the metalens with unit amplitude and phase map $\varphi_{k}(p, q)$, which creates the *k*-th focus on the image plane (**Fig. S25**). The transmittance function of the multifocal metalens using phase addition multiplexing can be expressed as $t_{mf,A}\left( p, q \right)=\frac{\sum_{k=1}^{n} e^{i\varphi_{k}}}{|\sum_{k=1}^{n} e^{i\varphi_{k}}|}=e^{i arg[\sum_{k=1}^{n} e^{i\varphi_{k}}]}$ where $n$ is the number of foci (**equation (1)** in results section). The subscript ‘$A$’ denotes phase addition multiplexing. According to Rayleigh–Sommerfeld propagation theory, the propagation of an optical field is described as follows^5,6^:

|  | $U(x,y)=\iint_{p, q} u\left( p, q \right)h\left( x-p, y-q \right)dpdq$ | (S4) |
| --- | --- | --- |
|  | $h\left( x, y, z \right)=\frac{z}{i\lambda}\frac{e^{iKr}}{r^{2}}, \mathrm{where} r=\sqrt{x^{2}+y^{2}+z^{2}}$ | (S5) |

where $K$ is the wavenumber, and $h$ is the impulse response of the Rayleigh-Sommerfeld equation. Thus, the optical field of a multifocal metalens using phase-addition multiplexing in the image plane can be expressed as follows:

|  | $U_{mf,A}\left( x, y \right)=\iint_{p, q} t_{mf,A}\left( p, q \right)h\left( x-p, y-q \right)dpdq$  $=\iint_{p, q} \left[ \frac{\sum_{k=1}^{n} t_{k}(p, q)}{\left\vert\sum_{k=1}^{n} t_{k}\left( p, q \right) \right\vert} \right]h\left( x-p, y-q \right)dpdq$ | (S6) |
| --- | --- | --- |

The normalization factor $|\sum_{k=1}^{n} t_{k}\left( p, q \right)|$ reaches its maximum value of $n$ when all phases are aligned ($\varphi_{1}=\varphi_{2}=\cdots=\varphi_{n}=\varphi, t_{k}=e^{i\varphi}$) at all $\left( p, q \right)$, meaning all the fields are constructively integrated. Therefore, $\left| \sum_{k=1}^{n} t_{k} \right|_{\max}=\left| ne^{i\varphi} \right|=n$. Conversely, the normalization factor reaches its minimum value of 0 when all the phases are evenly distributed (e.g., phases spaced by $2\pi/n$), leading to destructive interference, $\left| \sum_{k=1}^{n} t_{k} \right|_{\min}=0$.

|  | $\left\vert\sum_{k=1}^{n} t_{k}\left( p, q \right) \right\vert=\left\{ \begin{aligned} 0 \mathrm{if} \varphi_{k} are evenly distributed \\ 0<\left\vert\sum_{k=1}^{n} t_{k} \right\vert<n \mathrm{if} \varphi_{k} are partially aligned \\ n \mathrm{if} \varphi_{k} are perfectly aligned \end{aligned} \right.$ | (S7) |
| --- | --- | --- |

The intensity of the multifocal array $I_{mf,A}\left( x,y \right)=\left| U_{mf,A} \right|^{2}$ and its lower bound can be expressed as (**equation (S6)**):

|  | $I_{mf,A}\left( x,y \right)=\left\vert\iint_{p, q} \left[ \frac{\sum_{k=1}^{n} t_{k}\left( p, q \right)}{\left\vert\sum_{k=1}^{n} t_{k}\left( p, q \right) \right\vert} \right]h\left( x-p, y-q \right)dpdq \right\vert^{2}\geq\frac{1}{n^{2}}\left\vert\iint_{p, q} \left[ \sum_{k=1}^{n} t_{k}\left( p, q \right) \right]h\left( x-p, y-q \right)dpdq \right\vert^{2}$ | (S8) |
| --- | --- | --- |

The lower bound can be achieved when the phases $\varphi_{k}$ are perfectly aligned for all $k$ at any $\left( p, q \right)$, causing the denominator of $t_{mf,A}$ to become constant, $\left| \sum_{k=1}^{n} t_{k} \right|=n$ (**equation (S7)**).

Assuming a low NA for simplicity, where the focal length is much larger than the diameter of the metalens, the impulse response $h(x,y)$ can be approximated as:

|  | $h\left( x, y \right)=\frac{e^{iKr}}{i\lambda f}for low NA (f\gg D)$ | (S9) |
| --- | --- | --- |

where $D$ is the diameter of the metalens, and $f$ is the focal length (**equation (S5)**). This approximation assumes a propagation distance ($z$) equal to focal length ($f$), corresponding to projection onto the focal plane. The term $\left[ \sum_{k=1}^{n} t_{k}\left( p, q \right) \right]h\left( x-p, y-q \right)$ in **equation (S8)** can then be rewritten as $\frac{1}{i\lambda f}\sum_{k=1}^{n} e^{i(\varphi_{k}+Kr^{'})}$, where $r^{'}=\sqrt{{(x-p)}^{2}+{(y-q)}^{2}+z^{2}}$. Substituting this into **equation (S8)** gives the lower bound of the intensity $I_{mf,A, lb}=\frac{1}{n^{2}}\left| \frac{1}{i\lambda f}\iint_{p, q} \sum_{k=1}^{n} e^{i(\varphi_{k}+Kr^{'})}dpdq \right|^{2}$. Notably, the term $\varphi_{k}+Kr^{'}$ can be treated as constant with respect to $\left( p,q \right)$. This approximation holds because the metalens phase $\varphi_{k}=-\frac{2\pi}{\lambda} \left( \sqrt{\left( p-p_{0} \right)^{2}+\left( q-q_{0} \right)^{2}+f^{2}}-f \right)\approx0$ and $r^{'}\approx f$ for a large focal length (low NA), where $p_{0}$ and $q_{0}$ denote offset position. As a result, $\frac{1}{i\lambda f}\sum_{k=1}^{n} e^{i(\varphi_{k}+Kr^{'})}$ also can be treated as constant. Therefore, we can rewrite **equation (S8)** as follows:

|  | $I_{mf,A}\left( x,y \right)=\frac{1}{\left( \lambda f \right)^{2}}\left\vert\iint_{p, q} \frac{\sum_{k=1}^{n} U_{A,k}\left( x,y \right)}{\left\vert\sum_{k=1}^{n} t_{k}\left( p, q \right) \right\vert}dpdq \right\vert^{2}$  $\approx\frac{1}{\left( \lambda f \right)^{2}}\left\vert C\sum_{k=1}^{n} U_{A,k}\left( x,y \right) \right\vert^{2}$  $\geq I_{mf,A,lb}(x,y)=\frac{1}{\left( \lambda f \right)^{2}}\frac{A^{2}}{n^{2}}\left\vert\sum_{k=1}^{n} U_{A,k}(x,y) \right\vert^{2}$ | (S10) |
| --- | --- | --- |

where $A$ is the area of metalens, $U_{A,k}(x,y)=e^{i(\varphi_{k}+Kr^{'})}$, and $C=\iint_{p, q} \frac{1}{\left| \sum_{k=1}^{n} t_{k}\left( p, q \right) \right|}dpdq$. Notably, the $I_{mf, A,lb}$ correspond to the intensity of coherently superimposed fields $U_{A,k}(x,y)$ as discussed in **Supplementary Notes. 1**.

Now, consider the peak intensity of the *m*-th focus at $\left( x_{m}, y_{m} \right)$ in the image plane, which can be expressed with its lower bound as (**equation (S10)**):

|  | $I_{mf,A}\left( x_{m},y_{m} \right)=\frac{1}{\left( \lambda f \right)^{2}}\left\vert\iint_{p, q} \frac{\sum_{k=1}^{n} U_{A,k}(x_{m},y_{m})}{\left\vert\sum_{k=1}^{n} t_{k}\left( p, q \right) \right\vert}dpdq \right\vert^{2}$  $\geq I_{mf,A,lb}\left( x_{m},y_{m} \right)=\frac{1}{\left( \lambda f \right)^{2}}\frac{A^{2}}{n^{2}}\left\vert\sum_{k=1}^{n} U_{A,k}(x_{m},y_{m}) \right\vert^{2}$ | (S11) |
| --- | --- | --- |

If the multifocal array has a sufficiently large pitch to ignore interference with adjacent foci, we can treat the term $\left| \sum_{k=1}^{n} U_{A,k}(x_{m},y_{m}) \right|^{2}$ as incoherently superimposed. In this case, the lower bound becomes $I_{mf,A,lb}\left( x_{m},y_{m} \right)\approx\frac{1}{\left( \lambda f \right)^{2}}\frac{A^{2}}{n^{2}}\sum_{k=1}^{n} \left| U_{A,k}\left( x_{m},y_{m} \right) \right|^{2}$ (**equation (S11)**). In addition, the lower bound of the peak intensity of *m*-th focus can be further simplified as:

|  | $I_{mf,A,lb}\left( x_{m},y_{m} \right)\approx\frac{1}{\left( \lambda f \right)^{2}}\frac{A^{2}}{n^{2}}\left\vert U_{A,m}\left( x_{m},y_{m} \right) \right\vert^{2}$ | (S12) |
| --- | --- | --- |

because the electromagnetic energy at $\left( x_{m}, y_{m} \right)$ is primarily attributed to the phase profile of the *m*-th metalens, $e^{i\varphi_{m}}$. Although it is difficult to derive the exact solution for the peak intensity analytically, these approximate equations (**equation (S10)–(S12)**) provide valuable insights. The lower bound of the peak intensity is approximately proportional to the square of the metalens area and inversely proportional to the square of the number of foci. These trends are in good agreement with the simulation results, which demonstrate a degradation in focus quality as the number of foci increases or the diameter decreases (**Fig. 2c and Fig. S9**). Furthermore, the inhomogeneous peak intensity of a multifocal metalens designed using phase addition multiplexing with a small pitch can be explained by **equation (S8) and (S11)**. The equations describe how the scale factor $\left| \sum_{k=1}^{n} t_{k}\left( p, q \right) \right|$ is highly dependent on the coordinates and fields $U_{A,k}(x_{m},y_{m})$ in a complex manner, indicating that the interference significantly affects the peak intensity.

Supplementary Notes 3. Random multiplexing

The transmittance function of multifocal metalens using random multiplexing is given by $t_{mf,R}=e^{\sum_{k=1}^{n} i\varphi_{k}L_{k}}$, where $n$ is the number of foci, $\varphi_{k}(p, q)$ is the phase map creating *k*-th focus in the image plane, and $L_{k}(p, q)$ is a binary random matrix that satisfies $\sum_{k=1}^{n} L_{k}(p,q)=1 \forall(p,q)$. The subscript ‘$R$’ represents random multiplexing. It is assumed that the area of the region with a value of 1 for each binary random matrix $L_{k}$ is $\frac{A}{n}$ for any $k$, where $A$ is the area of the multifocal metalens. This indicates that the phase profiles of the multifocal metalens $\varphi_{k}\left( p, q \right)$ for all $k$ are combined with equal weighting factors.

The transmittance function of the multifocal metalens can be rewritten as follows^7^:

|  | $t_{mf,R}(p,q)=\sum_{k=1}^{n} e^{i\varphi_{k}(p,q)}L_{k}(p,q)$ | (S13) |
| --- | --- | --- |

For example, when $L_{m}\left( p^{'},q^{'} \right)=1$ at a certain coordinate $(p^{'},q^{'})$ and for a certain $m$, where $1\leq m\leq n$, all other $L_{k}\left( p^{'},q^{'} \right)=0$ where $k\neq m$ at $(p^{'},q^{'})$. Thus, $e^{\sum_{k=1}^{n} i\varphi_{k}(p^{'},q^{'})\times L_{k}(p^{'},q^{'})}=\sum_{k=1}^{n} e^{i\varphi_{k}\left( p^{'},q^{'} \right)}L_{k}(p',q')=e^{i\varphi_{m}(p^{'},q^{'})}$ at $(p^{'},q^{'})$ for $m$. Remarkably, unlike phase addition multiplexing method, there is no scale factor in the transmittance function when using random multiplexing. This is because $t_{mf,R}(p,q)$ is always reduced to $e^{i\varphi_{k}(p,q)}$ due to the random matrix term for a certain $k$, where $1\leq k\leq n$ at any $(p,q)$, resulting in $\left| t_{mf,R}(p,q) \right|=1$. Consequently, the optical field of the multifocal metalens in the image plane using random multiplexing method can be described by (**equation (S4) and (S13)**):

|  | $U_{mf,R}\left( x, y \right)=\iint_{p, q} \left[ \sum_{k=1}^{n} e^{i\varphi_{k}\left( p,q \right)}L_{k}\left( p,q \right) \right]h\left( x-p, y-q \right)dpdq$ | (S14) |
| --- | --- | --- |

Assuming low NA, the term $\left[ \sum_{k=1}^{n} e^{i\varphi_{k}\left( p,q \right)}L_{k}\left( p,q \right) \right]h\left( x-p, y-q \right)$ inside the integral can be simplified as $\frac{1}{i\lambda f}\sum_{k=1}^{n} L_{k}\left( p,q \right)e^{i(\varphi_{k}\left( p,q \right)+Kr^{'})}$ (**equation (S9) and (S14)**). Hence, the $U_{\mathrm{mf}}\left( x, y \right)$ can be rewritten as:

|  | $U_{mf,R}\left( x, y \right)\approx\frac{1}{i\lambda f}\iint_{p, q} \sum_{k=1}^{n} L_{k}\left( p,q \right)e^{i(\varphi_{k}+Kr^{'})}dpdq$ | (S15) |
| --- | --- | --- |

As mentioned previously (**Supplementary Notes. 2**) the term $e^{i(\varphi_{k}\left( p,q \right)+Kr^{'})}$ can be treated as constant with respect to the $(p, q)$. Additionally, the summation symbol can be moved outside the integral because $\sum_{k=1}^{n} e^{i\varphi_{k}\left( p^{'},q^{'} \right)}L_{k}(p^{'},q^{'})=e^{i\varphi_{m}(p^{'},q^{'})}$ at $(p^{'},q^{'})$ for a specific $m$, as previously noted. Therefore, $U_{mf,R}\left( x, y \right)\approx\frac{1}{i\lambda f}\sum_{k=1}^{n} \iint_{p, q} L_{k}\left( p,q \right)e^{i(\varphi_{k}+Kr^{'})}dpdq$. Notably, the term $\iint_{p, q} L_{k}\left( p,q \right)e^{i(\varphi_{k}+Kr^{'})}dpdq$ represents a weighted sum of $e^{i(\varphi_{k}+Kr^{'})}$, where the weighting factor is the area of the binary region in $L_{k}$ with a value of 1, corresponding to $\frac{A}{n}$. Hence, the $U_{mf,R}\left( x, y \right)$ can be rewritten as:

|  | $U_{mf,R}\left( x, y \right)\approx\frac{1}{i\lambda f}\frac{A}{n}\sum_{k=1}^{n} U_{R,k}\left( x,y \right)$ | (S16) |
| --- | --- | --- |

where $U_{R,k}(x,y)=e^{i(\varphi_{k}+Kr^{'})}$, and $\frac{A}{n}=\iint_{p, q} L_{k}\left( p,q \right)dpdq$ for any $k (1\leq k\leq n)$. The weighting factor $\frac{A}{n}$ is the same for all $k$, because the binary random matrices $L_{k}$ have an equal area ($\frac{A}{n}$). Therefore, the intensity of the multifocal array is given by:

|  | $I_{mf,R}\left( x,y \right)=\frac{1}{\left( \lambda f \right)^{2}}\frac{A^{2}}{n^{2}}\left\vert\sum_{k=1}^{n} U_{R,k}(x,y) \right\vert^{2}$ | (S17) |
| --- | --- | --- |

Remarkably, this is the same as the lower bound of the peak intensity for the multifocal metalens designed using phase-addition multiplexing (**equation (S10)**). By following the same process, a large pitch is assumed to ignore interference with adjacent foci. Because the energy in peak intensity of the *m*-th focus is mainly attributed to the phase profile of the *m*-th metalens, the peak intensity for random multiplexing is $I_{mf,R}\left( x_{m},y_{m} \right)=\frac{1}{\left( \lambda f \right)^{2}}\frac{A^{2}}{n^{2}}\left| U_{R,m}\left( x_{m},y_{m} \right) \right|^{2}$. The peak intensity was also proportional to the square of the metalens area and inversely proportional to the square of the number of foci, which agrees with previous reports^7,8^ and our simulation data (**Fig. 2c**).

By comparing **equation (S11)** and **(S17)**, we can explain why the beam quality (SNR or peak intensity) of the multifocal array designed using the phase addition method is better than that of the random matrix for a given number of foci (**Fig. 2c–d and Fig. S3–5, S7, and S8**). The peak intensity for the phase addition method is equal to or always higher than that for random multiplexing (**equation (S11) and (S17)**). Moreover, we can explain why the uniformity of the peak intensity for random multiplexing is better than that of phase addition methods with a small pitch, where interference is significant. In random multiplexing, the peak intensity is determined solely by the scale factor ($\frac{A^{2}}{n^{2}}$) and the superposition of the fields with uniform amplitude ($\left| \sum_{k=1}^{n} U_{R,k}(x_{m},y_{m}) \right|^{2}$) (**equation (S17)**). However, the peak intensity for phase addition multiplexing is more complex, $\left| \iint_{p, q} \frac{\sum_{k=1}^{n} U_{A,k}(x_{m},y_{m})}{\left| \sum_{k=1}^{n} t_{k}\left( p, q \right) \right|}dpdq \right|^{2}$ (**equation (S11)**), which depends not only on the superposition of the fields, but also on the scale factor $\left| \sum_{k=1}^{n} t_{k}\left( p, q \right) \right|$. Consequently, phase addition multiplexing has an advantage in beam quality but is more susceptible to variations in peak intensity uniformity, particularly at small pitches. In contrast, random multiplexing offers better uniformity in peak intensity but is limited in its ability to increase the number of foci, as this leads to a degradation in the beam quality.

Supplementary Notes 4. Hybrid multiplexing

For hybrid multiplexing, the transmittance function of a multifocal metalens is given by $t_{mf,H}=e^{i\left( \psi_{e}L_{1}+\psi_{o}L_{2} \right)}$, where the subscript ‘$H$’ denotes the hybrid multiplexing, and $L_{1}\left( p,q \right)$ and $L_{2}\left( p, q \right)$ are binary random matrices that satisfy $L_{1}\left( p, q \right)+ L_{2}\left( p, q \right)=1$ for all $\left( p, q \right)$ (**equation (5) and (6)** in results section). The terms $\psi_{e}$ and $\psi_{o}$, which generate mutually interleaved multifocal arrays, are described as follows:

|  | $\psi\left( p,q \right)=arg\left[ \sum_{k+r=2}^{n} e^{i\varphi_{k,r}\left( p,q \right)} \right]$  ,$\mathrm{where}$  $\psi\left( p,q \right)=\left\{ \begin{aligned} \psi_{e}\left( p,q \right) \mathrm{if} \left( k+r \right) is even \\ \psi_{o}\left( p,q \right) \mathrm{if} \left( k+r \right) is odd \end{aligned} \right., k, r are positive integers$ | (S18) |
| --- | --- | --- |

where $\varphi_{k,r}(p, q)$ represents the phase map of a single metalens, and $n$ is the number of foci. The subscripts ‘$k$’ and ‘$r$’ represent the column and row indices, respectively, for the foci arranged in a grid pattern. Following the same procedure as in **equation (S13)**, the transmittance function can be described as $t_{mf,H}=e^{i\psi_{e}}L_{1}+e^{i\psi_{o}}L_{2}$. Furthermore, this can be rewritten as follows:

|  | $t_{mf,H}\left( p, q \right)=\frac{\sum_{k+r=2}^{n} e^{i\varphi_{k,r, e}}}{\vert\sum_{k+r=2}^{n} e^{i\varphi_{k,r, e}}\vert}L_{1}+\frac{\sum_{k+r=2}^{n} e^{i\varphi_{k,r, o}}}{\vert\sum_{k+r=2}^{n} e^{i\varphi_{k,r, o}}\vert}L_{2}$  $, where \varphi_{k,r}\left( p,q \right)=\left\{ \begin{aligned} \varphi_{k,r,e} \mathrm{if}k+r is even \\ \varphi_{k,r,o} \mathrm{if} k+r is odd \end{aligned} \right.$ | (S19) |
| --- | --- | --- |

By substituting **equation (S19)** into **equation (S4)**, the optical field of the multifocal metalens using hybrid multiplexing in the image plane can be expressed as:

|  | $U_{mf,H}\left( x, y \right)=$  $\iint_{p, q} \left[ \frac{\sum_{k+r=2}^{n} e^{i\varphi_{k,r,e}}}{\vert\sum_{k+r=2}^{n} e^{i\varphi_{k,r, e}}\vert}L_{1}+\frac{\sum_{k+r=2}^{n} e^{i\varphi_{k,r, o}}}{\vert\sum_{k+r=2}^{n} e^{i\varphi_{k,r, o}}\vert}L_{2} \right]h\left( x-p, y-q \right)dpdq$ | (S20) |
| --- | --- | --- |

Additionally, the intensity of the multifocal array, including its lower bound, can be written as:

|  | $I_{mf,H}\left( x,y \right)=$  $\left\vert\iint_{p, q} \left[ \frac{\sum_{k+r=2}^{n} e^{i\varphi_{k,r,e}}}{\left\vert\sum_{k+r=2}^{n} e^{i\varphi_{k,r, e}} \right\vert}L_{1}+\frac{\sum_{k+r=2}^{n} e^{i\varphi_{k,r, o}}}{\left\vert\sum_{k+r=2}^{n} e^{i\varphi_{k,r, o}} \right\vert}L_{2} \right]h\left( x-p, y-q \right)dpdq \right\vert^{2}$  $\geq\frac{4}{n^{2}}\left\vert\iint_{p, q} \left[ \sum_{k+r=2}^{n} e^{i\varphi_{k,r,e}}L_{1}+\sum_{k+r=2}^{n} e^{i\varphi_{k,r, o}}L_{2} \right]h\left( x-p, y-q \right)dpdq \right\vert^{2}$ | (S21) |
| --- | --- | --- |

The lower bound is achieved when $\sum_{k+r=2}^{n} e^{i\varphi_{k,r, e}}$ and $\sum_{k+r=2}^{n} e^{i\varphi_{k,r, o}}$ are perfectly aligned. The maximum value for each normalization factors is $\left| \sum_{k+r=2}^{n} e^{i\varphi_{k,r, e}} \right|_{\max}=\left| \sum_{k+r=2}^{n} e^{i\varphi_{k,r, o}} \right|_{\max}=\frac{n}{2}$ because the number of terms in each normalization factor is half of the number of total foci ($\frac{n}{2}$). By repeating the assumption of a low NA, the intensity can be simplified as:

|  | $I_{mf,H}\left( x,y \right)=$  $=\frac{1}{\left( \lambda f \right)^{2}}\left\vert\iint_{p, q} \left[ \frac{\sum_{k+r=2}^{n} U_{H,e}^{k,r}}{\left\vert\sum_{k+r=2}^{n} e^{i\varphi_{k,r, e}} \right\vert}L_{1}+\frac{\sum_{k+r=2}^{n} U_{H,o}^{k,r}}{\left\vert\sum_{k+r=2}^{n} e^{i\varphi_{k,r, o}} \right\vert}L_{2} \right]dpdq \right\vert^{2}$  $\approx\frac{1}{\left( \lambda f \right)^{2}}\left\vert C_{1}\sum_{k+r=2}^{n} U_{H,e}^{k,r}+C_{2}\sum_{k+r=2}^{n} U_{H,o}^{k,r} \right\vert^{2}$  $\geq I_{mf,H,lb}\left( x, y \right)=\frac{1}{\left( \lambda f \right)^{2}}\frac{A^{2}}{n^{2}}\left\vert\sum_{k+r=2}^{n} U_{H,e}^{k,r}+\sum_{k+r=2}^{n} U_{H,o}^{k,r} \right\vert^{2}$ | (S22) |
| --- | --- | --- |
|  | $U_{H,e}^{k,r}\left( x, y \right)=e^{i\left( \varphi_{k,r,e}+Kr^{'} \right)}$  $U_{H,o}^{k,r}(x, y)=e^{i(\varphi_{k,r,o}+Kr^{'})}$ | (S23) |

where $C_{1}=\iint_{p, q} \frac{L_{1}}{\left| \sum_{k+r=2}^{n} e^{i\varphi_{k,r, e}} \right|}dpdq$ and $C_{2}=\iint_{p, q} \frac{L_{2}}{\left| \sum_{k+r=2}^{n} e^{i\varphi_{k,r, o}} \right|}dpdq$. For the lower bound, the weighting factor related to the area of the binary random matrices is $\frac{A}{2}$, because only two random matrices are used for hybrid multiplexing. Remarkably, this lower bound ($I_{mf,H,lb}$) is essentially the same as the intensity lower bound for the phase addition method ($I_{mf,A,lb}$) (**equation (S10)**). Finally, the peak intensity of *m*-th focus is as follows:

|  | $I_{mf,H}\left( x_{m},y_{m} \right)$  $=\frac{1}{\left( \lambda f \right)^{2}}\left\vert\iint_{p, q} \left[ \frac{\sum_{k+r=2}^{n} U_{H,m,e}^{k,r}}{\left\vert\sum_{k+r=2}^{n} e^{i\varphi_{k,r, e}} \right\vert}L_{1}+\frac{\sum_{k+r=2}^{n} U_{H,m,o}^{k,r}}{\left\vert\sum_{k+r=2}^{n} e^{i\varphi_{k,r, o}} \right\vert}L_{2} \right]dpdq \right\vert^{2}$  $\geq I_{mf,H,lb}\left( x_{m}, y_{m} \right)=\frac{1}{\left( \lambda f \right)^{2}}\frac{A^{2}}{n^{2}}\left\vert\sum_{k+r=2}^{n} U_{H,m,e}^{k,r}+\sum_{k+r=2}^{n} U_{H,m,o}^{k,r} \right\vert^{2}$ | (S24) |
| --- | --- | --- |
|  | $U_{H,m,e}^{k,r}=U_{H,e}^{k,r}\left( x_{m}, y_{m} \right)$  $U_{H,m,o}^{k,r}=U_{H,o}^{k,r}(x_{m}, y_{m})$ | (S25) |

If the peak intensity of the *m*-th focus corresponds to one of the even foci, the peak intensity can be simplified as $I_{mf,H}\left( x_{m},y_{m} \right)\approx\frac{1}{\left( \lambda f \right)^{2}}\left| \iint_{p, q} \frac{\sum_{k+r=2}^{n} U_{H,m,e}^{k,r}}{\left| \sum_{k+r=2}^{n} e^{i\varphi_{k,r, e}} \right|}L_{1}dpdq \right|^{2}$, because the energy at the *m*-th foci mainly originates from the first term (**equation (S24)**). In fact, the peak intensity at $\left( x_{m},y_{m} \right)$ is the area-weighted sum of fields across all $\left( p,q \right)$. However, because of the nature of the binary random matrix, fields corresponding to the even and odd phase profiles cannot simultaneously contribute to $I_{mf,H}\left( x_{m},y_{m} \right)$ at any given $\left( p,q \right)$. Therefore, only the first term (for even foci) or second term (for odd foci) is valid. Consequently, interactions are limited to even phase profiles in the case of even foci, which leads to reduced interference and results in a more homogeneous peak intensity compared with the phase addition method.

# **Reference**

1. Kintner, E. C. & Sillitto, R. M. Two-point Resolution Criteria in Partially Coherent Imaging. *Opt. Acta Int. J. Opt.* **20**, 721–728 (1973).

2. Sparrow, C. M. On spectroscopic resolving power. *Astrophys. J. Vol 44 P 76* **44**, 76 (1916).

3. Magana-Loaiza, O. S. *et al.* Exotic looped trajectories of photons in three-slit interference. *Nat. Commun.* **7**, 13987 (2016).

4. Sinha, A., H. Vijay, A. & Sinha, U. On the superposition principle in interference experiments. *Sci. Rep.* **5**, 10304 (2015).

5. Voelz, D. G. Computational fourier optics: a MATLAB tutorial. *No Title* 51 (2011).

6. Goodman, J. W. *Introduction to Fourier Optics*. (Roberts and Company publishers, 2005).

7. Zhao, J. *et al.* Rapid Cellular-Resolution Skin Imaging with Optical Coherence Tomography Using All-Glass Multifocal Metasurfaces. *ACS Nano* **17**, 3442–3451 (2023).

8. Lin, D. *et al.* Photonic Multitasking Interleaved Si Nanoantenna Phased Array. *Nano Lett.* **16**, 7671–7676 (2016).
